# Microbes of biotechnological importance in acidic saline lakes in the Yilgarn Craton, Western Australia

**DOI:** 10.3389/fmicb.2024.1308797

**Published:** 2024-02-14

**Authors:** Katelyn Boase, Talitha Santini, Elizabeth Watkin

**Affiliations:** ^1^Curtin Medical School, Curtin University, Perth, WA, Australia; ^2^School of Agriculture, University of Western Australia, Perth, WA, Australia; ^3^School of Science, Edith Cowan University, Perth, WA, Australia

**Keywords:** Yilgarn Craton, microbial ecology, extremophiles, acidophiles, halophiles, biotechnology

## Abstract

Acidic salt lakes are environments that harbor an array of biologically challenging conditions. Through 16S rRNA, 18S rRNA, and ITS amplicon sequencing of eight such lakes across the Yilgarn Craton of Western Australia, we aim to understand the microbial ecology of these lakes with a focus on iron- and sulfur-oxidizing and reducing microorganisms that have theoretical application in biomining industries. In spite of the biological challenges to life in these lakes, the microbial communities were highly diverse. Redundancy analysis of soil samples revealed sulfur, ammonium, organic carbon, and potassium were significant diversities of the microbial community composition. The most abundant microbes with a hypothetical application in biomining include the genus *9 M32* of the *Acidithiobacillus* family, *Alicyclobacillus* and *Acidiphilium*, all of which are possible iron- and/or sulfur-oxidizing bacteria. It is evident through this study that these lakes harbor multiple organisms with potential in biomining industries that should be exploited and studied further.

## 1 Introduction

The Yilgarn Craton constitutes a substantial portion of Western Australia’s land mass and is composed of approximately 2.8 billion-year-old granite-gneiss metamorphic terrain and granite-greenstone terrains. The craton is divided into a series of provinces; our samples were acquired from the Youanmi Terrane and the South West Terrane (Cassidy et al., 2006). Salinity found in this region has occurred through natural processes due to the flatness of the landscape, the historical magnitude of weathering, low rainfall, and high evaporation (George et al., 2008). Further salinization of the area has occurred due to the clearing of native flora, resulting in a rising water table and an increase in the discharge of saline groundwater into surface streams (Hatton et al., 2003). As a result of this salinization, the Yilgarn Craton contains hundreds of ephemeral salt lakes. Year-round, these lakes are exposed to high levels of UV radiation, flooding, evaporation, and low pH levels. The Yilgarn Craton typically has acidic groundwaters as low as pH 1.4, hypothesized to be the result of the oxidation of sulfides from abundant sulfide minerals present (Benison and Bowen, 2015). Other acidification is likely due to the ferrolysis of waters high in iron and aluminum, acidophilic bacteria further oxidizing minerals and releasing protons, evaporation resulting in the concentration of protons, and the natural lack of geochemical buffers (carbonate minerals; Bowen and Benison, 2009; Benison and Bowen, 2015).

Naturally occurring environments both high in salinity and acidity are rare, which is likely why only a small amount of haloacidophiles have been characterized. Additionally, acidophiles are notorious for being highly sensitive to chloride ions, possibly another biological barrier to the evolution of haloacidophiles (Suzuki et al., 1999; Baker-Austin and Dopson, 2007). Previous research involving the biology of acidic saline environments has been motivated by isolating iron- and sulfur-oxidizing bacteria that have potential in saline biomining operations as well as understanding the biological basis of acid and saline polyextremophiles (Simmons and Norris, 2002; Zammit et al., 2009; O’Dell et al., 2018; Chen and Schlömann, 2023; Corbett and Watkin, 2023). Using haloacidophilic iron- and sulfur-oxidizing microbes for bioleaching operations has the possibility to reduce the cost of biomining as seawater may be able to replace reverse osmosis water currently used to irrigate these processes (Rohwerder et al., 2003; Rea et al., 2015; Johnson and Schippers, 2017). There is currently a large range of microbes that have been identified for their ability to leach minerals through iron and/or sulfide oxidation in mining contexts, for example, genera such as *Acidithiobacillus*, *Acidimicrobium*, *Acidiphilium*, *Leptospirillum*, *Alicyclobacillus*, *Ferroplasma*, *Sulfolobus*, and *Acidihalobacter* (Gumulya et al., 2018; Roberto and Schippers, 2022). Although iron- and sulfur-oxidizing bacteria are important in this context, iron-reducing microbes also have applications in leaching commercially important metals. Reductive dissolution of oxidized mineral ores allows the further leaching of metals from oxidized minerals that are normally considered waste (Johnson, 2018). Genera that have been identified in this context include many of the iron-oxidizing microbes already mentioned, as they are able to reduce iron in anaerobic conditions (Hallberg et al., 2011; Marrero et al., 2015). Given the biotechnological use of these microbes, it is important to explore their natural habitats. Although many studies have identified such genera in a wide variety of natural habitats, some of which are in Western Australia, no such study has sampled a multitude of lakes and sample types (soil, salt brine, and water). Thus, this study aims to describe and explore the microbial ecology of eight extremely acidic ephemeral lakes in the Yilgarn Craton which may inform future bioprospecting for the isolation of haloacidophilic iron- and sulfur-oxidizing/reducing microbes for potential application in biomining and to understand what geochemical conditions drive these microbial communities.

## 2 Materials and methods

### 2.1 Sample collection

**Figure 1:**
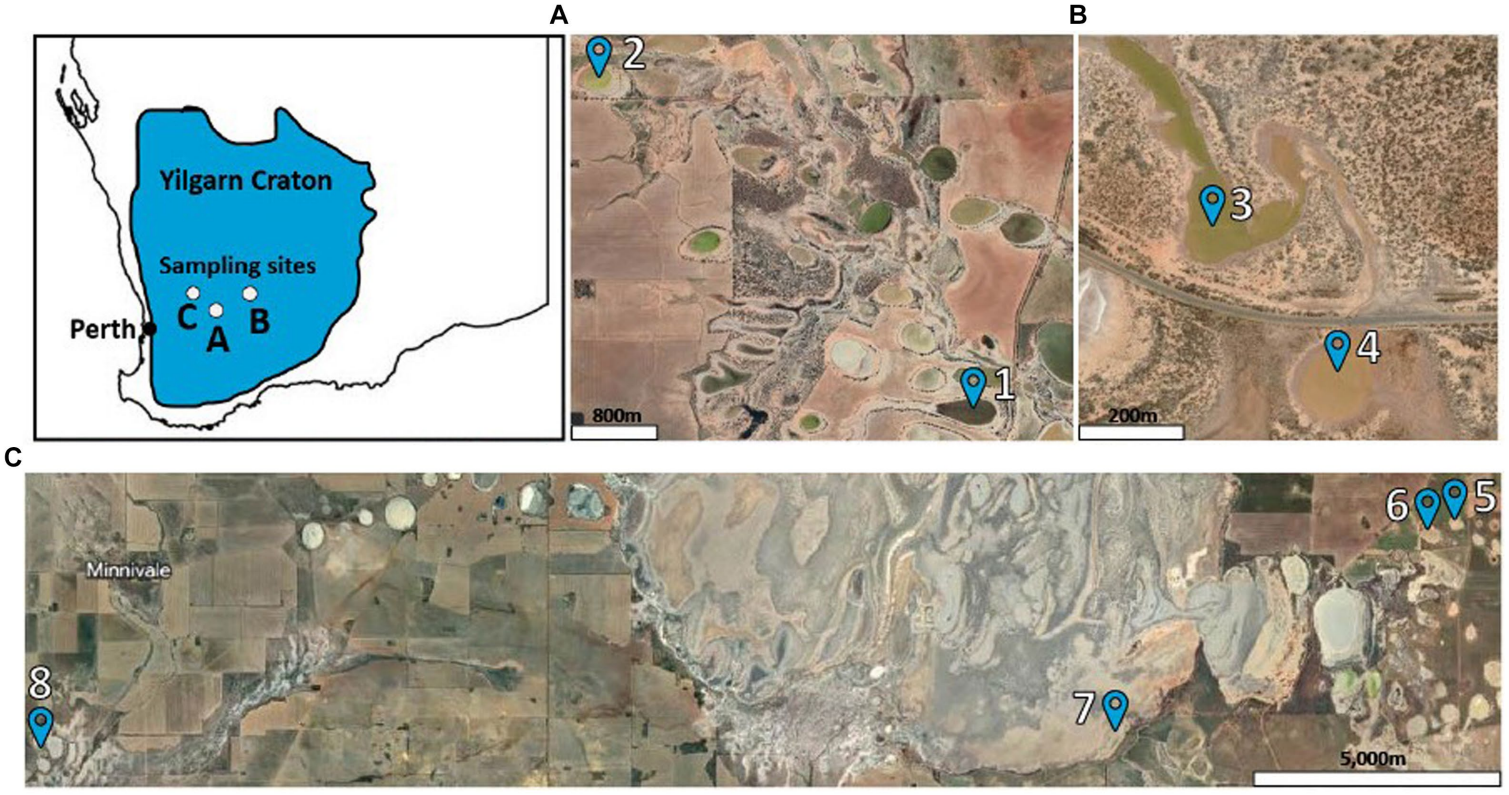
Lake sampling locations. Site **(A)** included Lakes 1 and 2, site **(B)** included Lakes 3 and 4, and site **(C)** included Lakes 5, 6, 7, and 8.

Sample sites were screened using data provided by the Western Australian Department of Water and Environmental Regulation. A total of 8 lakes were chosen for the study that were reported to have a pH below 4 and salinity levels of 35,000 TDS and above. Locations of the lakes can be seen in Figure 1, and coordinates can be viewed in Supplementary Table 1. Sample collection followed the Australian Microbiome soil and water collection protocol^1^ over a 3-day period starting on 8 June 2020. From each lake, samples were collected from soil at 0–10 cm and 20–30 cm, salt brine, and lake water. At each lake, samples were collected as follows: 5 L of water from the center of the lake was filtered through a 0.2-μm filter, followed by a 0.1-μm filter. Thirty soil samples were collected within a 25-m^2^ area that spanned the shore of the lake to encompass the most environmental variability. Soil was taken at two depths (0–10 and 20–30 cm). The 30 samples from the same depth profile were homogenized in sterile whirl packs. Salt brine samples were collected by carefully scraping salt brine from the surface of the lake soil and homogenized in a sterile whirl pack. All 32 samples were stored in liquid nitrogen on-site and in −80°C freezers when returned to the laboratory. Additional water, soil, and salt brine samples were stored at room temperature for chemical analysis. Additional details about the samples collected can be found in Supplementary Table 1.

### 2.2 Chemical analysis of water and soil samples

Soil and water samples were sent to the CSBP Soil and Plant Analysis Laboratory for chemical analysis. A detailed description of the methods used can be viewed on the CSBP website (soil methods: https://www.csbplab.com.au/tests/soil/soil-comprehensive, water methods: https://www.csbplab.com.au/tests/water/water-standard). In summary, water samples were tested for trace elements (phosphorus, potassium, sulfur, copper, manganese, sodium, iron, and boron) using inductively coupled plasma (ICP) spectroscopy; nitrate nitrogen was measured by reducing nitrate to nitrite via a copperized cadmium column and measured colorimetrically; ammonium nitrogen and chloride were measured colorimetrically; pH was measured by a pH probe; electrical conductivity was measured by a conductivity probe; and carbonate was measured by titration against HCl. The soil and salt brine samples were analyzed using the following methods; phosphorus and potassium using the Colwell method (Colwell, 1965); sulfur was measured as described in Blair et al. (1991); organic carbon was measured as described in Walkley and Armstrong Black (1934); nitrate nitrogen and ammonium nitrogen was extracted using 2 M KCl and measured as described in the water analysis; for electrical conductivity and pH the soil is was mixed with deionized water for an hour and measured using a pH and electrical conductivity probe, additionally CaCl is added to the mixture to 0.01 M and pH is measured with a pH probe; boron was measured by extracting soil using 0.01 M CaCl_2_ in a 1:4 ratio, heated to 90°C, and measured using ICP spectroscopy; trace elements (copper, zinc, manganese, and iron) were measured using diethylene-triamine-penta-acetic acid (DTPA) solution (1:2 ratio with the soil) for 2 h and measured using atomic absorption spectroscopy; calcium, magnesium, sodium, potassium, and aluminum were measured using a mixture of 0.1 M ammonium chloride and barium chloride (ratio 1:10) and measured using ICP spectroscopy.

### 2.3 DNA extraction and sequencing

DNA was extracted in triplicate from all 32 samples (0–10 cm soil, 20–30 cm soil, water, and salt brine for each of the eight lakes) using the following method: salt brine and soil samples were extracted using the FastDNA™ Spin Kit for Soil, using approximately 500 mg of sample. Alterations to the extraction protocol are as follows: 40 mg of sterilized skim milk powder was added to soil and salt brine samples. Prior to ribolyzing, the samples were subjected to three cycles of freeze-thawing in liquid nitrogen and a 95°C heat block. Ribolyzing was performed in triplicate at 6.5 m/s for 40 s in a Fast-Prep24™ ribolyzer with a 5-min icing stage between the ribolyzing. Water filter extractions followed the same protocol but did not include the addition of skim milk powder. Filter samples were cut into small pieces using a sterilized scalpel and added to the kit.

Triplicate DNA extractions were combined for sequencing at the Ramaciotti Centre for Genomics (Sydney, Australia). The bacterial 16S rRNA V1-V3 region was amplified using 27F-519R primers; the archaeal 16S rRNA V1-V3 region was amplified using A2F-519R primers; and the eukaryotic 18S rRNA V4 region was amplified using the forward primer CCAGCASCYGCGGTAATTCC and the reverse primer ACTTTCGTTCTTGATYRATGA. Fungal ITS gene sequences were amplified using the ITS1 forward and ITS4 reverse primers. All amplicons were sequenced using the Illumina MiSeq paired-end platform. Additional details regarding the sequencing and library preparation can be accessed through the official Australian Microbiome Initiative manual at https://www.australianmicrobiome.com/protocols/. All sequence data are available at the NCBI Sequence Read Archive, Bioproject PRJNA1037708.

### 2.4 Bioinformatic and statistical analysis

The quality of the amplicons was assessed using both FastQC^2^ and MultiQC (Ewels et al., 2016). Trimming, filtering, denoising, and taxonomic assignment of amplicon sequence variants (ASVs) was done using DADA2 version 1.29.0 following recommendations in the DADA2 protocols (Callahan et al., 2016). Contaminant sequences were removed using the Microdecon package version 1.2.0 (McKnight et al., 2019). Rarefication was done using Vegan version 2.6–4 (Oksanen et al., 2022). Additional detailed pre-processing information and rarefication curves can be found in Supplementary methods and Supplementary Figures 1–4.

In-house scripts were used to calculate the alpha diversity measures in R. Redundancy analysis (RDA) and Spearman’s correlation were used to evaluate the correlation of the environmental data to the microbial community matrix. RDA was conducted using the vegan::rda application, and significance testing of the RDA was done via PERMANOVA. The number of water samples was insufficient to create significant RDAs. Spearman’s correlation was done using rcor() from the Hmisc R package (Harrell, 2023). The Vegan package was used to create non-metric ordination (NMDS) plots using metaMDS on a Bray–Curtis dissimilarity matrix of the microbial data. ggplot2 was used to format all plots for presentation (Wickham, 2016).

## 3 Results

### 3.1 Bacterial and archaeal community composition

**Figure 2:**
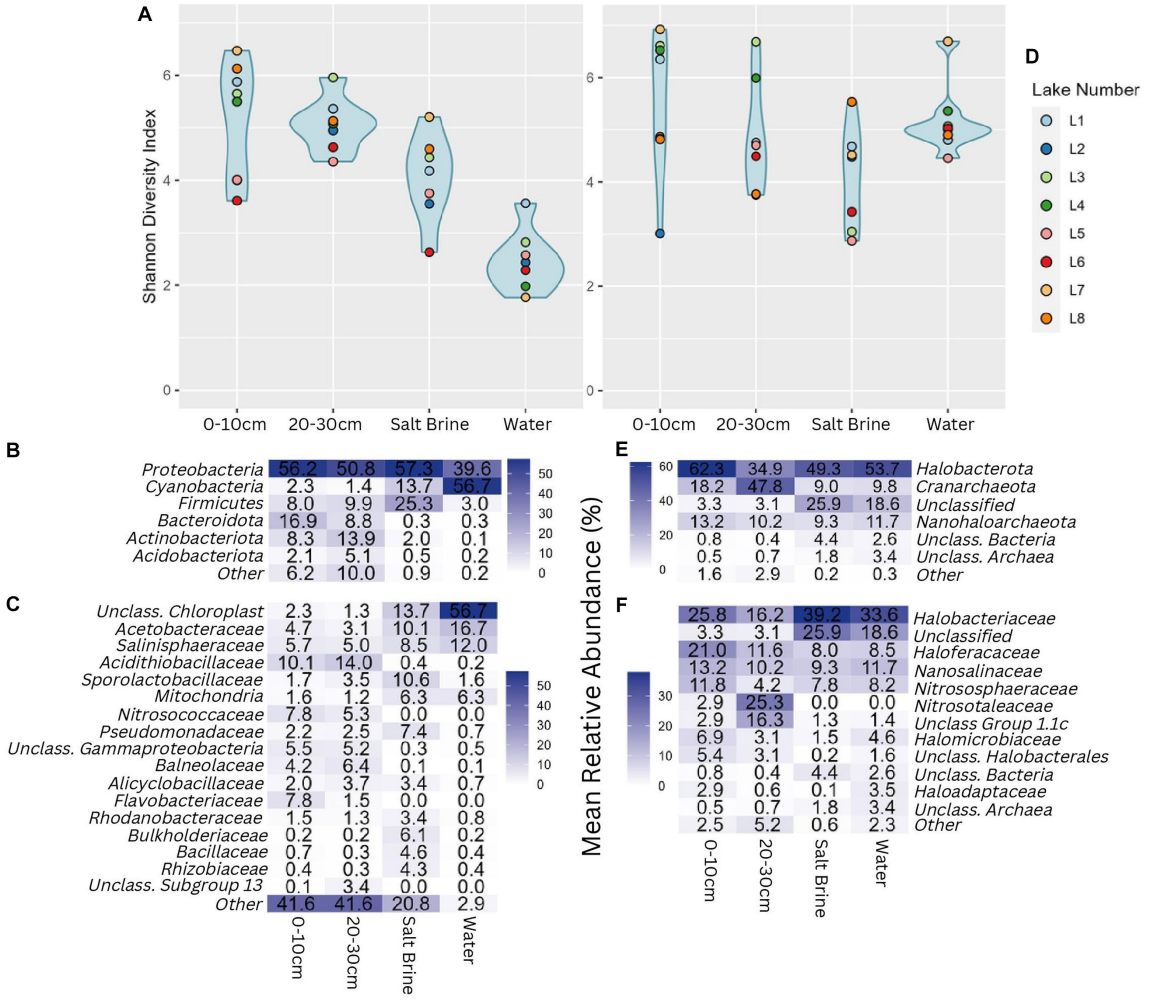
**(A)** Shannon diversity index of the bacterial community grouped by sample type; blue shading represents the distribution of the values plotted. **(B)** Percentage abundance heatmap of the bacterial phyla grouped by sample type. **(C)** Percentage abundance heatmap of the bacterial families grouped by sample type. **(D)** Shannon diversity index of the archaeal community grouped by sample type. **(E)** Percentage abundance heatmap of the archaeal phyla grouped by sample type. **(F)** Percentage abundance heatmap of the archaeal families grouped by sample type. Taxonomic levels that constituted less than 3% of any sample type were represented under other.

**Figure 3:**
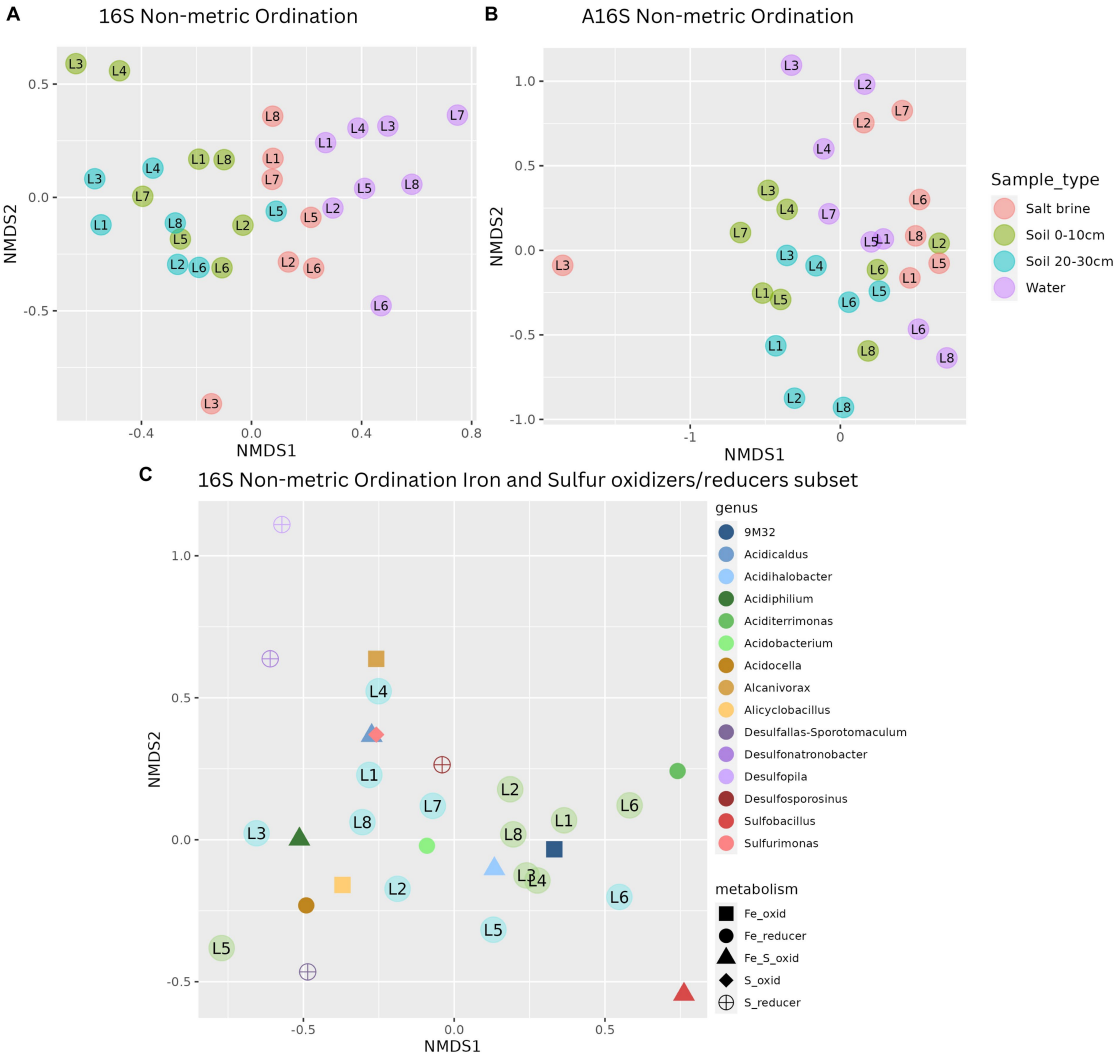
**(A)** Non-metric ordination of bacterial microbial communities with a stress score of 0.188. **(B)** Non-metric ordination of archaeal microbial communities with a stress score of 0.21. **(C)** Non-metric ordination of the known bacterial iron and sulfur oxidizers/reducers, stress score 0.117, soil sample sites at a depth of 0–10 cm in blue, and soil sample sites at a depth of 20–30 cm in green.

Alpha diversity measures of the bacterial communities indicate soil samples were more diverse on average when compared to salt brine and water samples (Figure 2A). The archaeal community had a wider Shannon index within the sample types compared to the bacterial community, and across the board, it had higher Shannon indices (Figure 2D). Lake 7 had the highest bacterial alpha diversity in the 0–10 cm soil samples and salt brine samples; Lake 3 had the highest diversity in the 20–30 cm depth; and Lake 1 had the highest diversity in the water samples. Lakes 5 and 6 generally had the lowest Shannon diversity indices in both the bacterial soil samples, and Lake 2 had low diversity in the archaeal 0–10 cm soil samples. *Proteobacteria* was the most abundant bacterial phyla across all sample types, excluding the water samples, which had the highest abundance of *Cyanobacteria* (Figure 2B). *Bacteroidota* and *Actinobacteriota* were abundant in the soil samples, whereas *Firmicutes* was abundant in the salt brine samples. When looking at the family level, 41% of the soil microbiota were classified as families with abundances below 3% (across all sample types), thus falling under the “Other” category (Figure 2C). The *Acidithiobacilliaceae* family was the most abundant bacterial family in the soil samples. Halophiles and thermophiles: *Halobacterota*, *Crenarchaeota*, and *Nanohaloarcheota* were the most abundant archaeal phyla across all sample types (Figure 2E). *Halobacteriaceae*, *Haloferaceae*, *Nanosalinaceae*, and *Nitrosphaeraceae* were the most abundant families across all sample types in the archaeal dataset (Figure 2F). The soil samples at 20–30 cm were the only sample type to have a high abundance of *Nitrosotaleaceae* and the Unclassified Group 1.1c family. Non-metric ordination plots revealed that the bacterial and microbial communities clustered based on sample type rather than location (Figure 3A). The soil samples at different depths clustered together on the ordination, which makes it less clear if the microbial communities at the different depths form distinct microbial communities. The ordination of the archaeal dataset does not show obvious clustering of sample types as seen for the bacterial dataset (Figure 3B). Lake 3 salt brine is plotted much further away from the remaining samples, suggesting its microbial community is quite different from all others.

Redundancy analysis of the soil and salt samples reveals that the bacterial diversity of many of the soil samples at both depths was positively correlated with potassium and conductivity and negatively related to pH, calcium, iron, and sulfur (Supplementary Figure 5). The bacterial diversity seen in 20–30 cm soil samples and salt brine samples of Lake 5 was positively correlated with sodium and negatively correlated with manganese ammonium and copper, whereas the diversity observed in all 20–30 cm soil samples, with the exception of Lake 5, is positively correlated with copper, conductivity, and potassium. The 0–10 cm soil samples are the most widely distributed sample type, suggesting these samples had less predictable environmental influences that drove the microbial community. Redundancy analysis of the archaeal soil and salt brine samples shows a distinct separation of salt brine samples from the soil samples, indicating the archaeal salt brine samples were driven by increased sodium, magnesium, and aluminum concentrations (Supplementary Figure 6).

A subset of known iron- and sulfur-oxidizing and reducing bacteria that have been previously identified in biomining contexts was created to obtain closer insight into these communities in the lakes sampled in this study. Non-metric ordination of this community reveals the most abundant iron oxidizer of the *Acidithiobacillus* family (9 M32 spp.), the iron- and sulfur-oxidizing haloacidophile *Acidihalobacter* spp., and the iron-reducing *Aciditerrimonas* spp. were closely associated with the 0–10 cm soil samples (Figure 3C). The remaining iron- and sulfur-oxidizing and reducing organisms were related to the 20–30 cm soil samples. Iron oxidizers, based on abundance data, were the most represented type of organism in the lakes, followed by sulfur oxidizers, sulfur reducers, and iron reducers, respectively. Spearman’s correlation of the same subset of genera in the soil and salt brine samples to the environmental data revealed significant positive correlations between aluminum concentrations and *Sulfobacillus*, iron and *Desulfosporosinus*, *Desulfopila*, and *Acidocella*, sodium, ammonium, and organic carbon with *Desulfopila*, and pH with *Alanivorax* and *Acidicaldus*. Significant negative correlations were reported between aluminum and *Alcanivorax*, boron and conductivity with *Acidiphilium* and *Acidobacterium*, copper with *Acidobacterium* and *Alicyclobacillus*, potassium with *Acidicaldus* and *Sulfurimonas*, magnesium with *Alicyclobacillus*, *Acidobacterium*, and *Acidiphilium*, manganese with 9 M32, *Acidobacterium* and *Acidiphilium*, sodium and *Acidobacterium*, pH with *9 M32*, *Sulfobacillus*, and *Acidihalobacter*. These results can be viewed in Supplementary Figure 7.

### 3.2 Fungal and eukaryotic community composition

**Figure 4:**
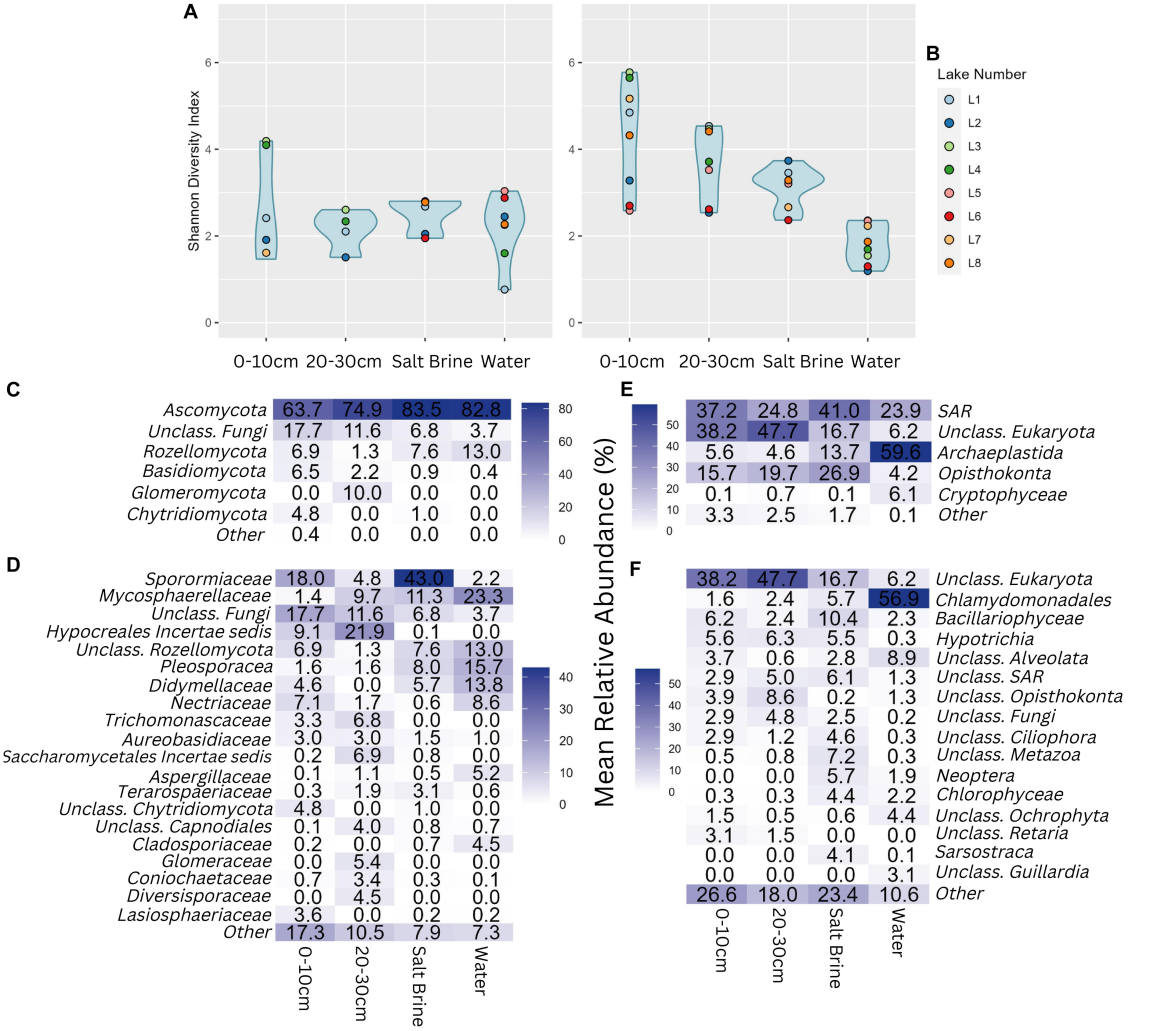
**(A)** Shannon diversity index of the fungal community grouped by sample type. **(B)** Shannon diversity index of the eukaryotic community, grouped by sample type. **(C)** Percentage abundance heatmap of the fungal phyla grouped by sample type. **(D)** Percentage abundance heatmap of the fungal families grouped by sample type. **(E)** Percentage abundance heatmap of the eukaryotic phyla grouped by sample type. **(F)** Percentage abundance heatmap of the eukaryotic families grouped by sample type. Taxonomic levels that constituted less than 4% for ITS and 3% for 18S of any sample type were represented under Other.

Alpha diversity of the fungal community shows much less variation than the bacterial and archaeal alpha diversity measures. Almost all samples have similar alpha diversity results, apart from the 0–10 cm soil samples of Lakes 3 and 4, which had a higher Shannon diversity index (Figure 4A). Alpha diversity measures of the eukaryotic community follow a similar trend as the bacterial and archaeal datasets, with the water samples having the lowest microbial diversity, followed by salt brine, 20–30 cm soil, and 0–10 cm soil samples with the highest average diversity (Figure 4B). Lakes 6 and 2 consistently had the lowest alpha diversity measures in comparison to the remaining samples, with the exception of the fungal water communities and the eukaryotic salt brine community of Lake 2. In terms of fungal phyla, all sample types were dominated by *Ascomycota*; the soil samples had the largest abundance of fungi that could not be classified past the phyla level (Figure 4C). The 20–30 cm soil samples were the only samples that had the *Glomeromycota* phylum present. In terms of family-level fungal classification, *Sporomiaceae*, *Mycospaerellaceae*, *Hypocreales incertae sedis*, *Pleosporaceae*, and *Didymellaceae* were the most abundant (Figure 4D). The majority of ASVs in the soil samples were assigned as “Unclassified Eukaryota,” highlighting how much of the eukaryotic dataset is unknown (Figure 4E). The phylum *Archaeplastids* was dominant in the water samples (which mostly consisted of the *Chlamydomondales* family, Figure 4F), and *Opisthokonta* was mostly present in the soil and salt brine samples.

**Figure 5:**
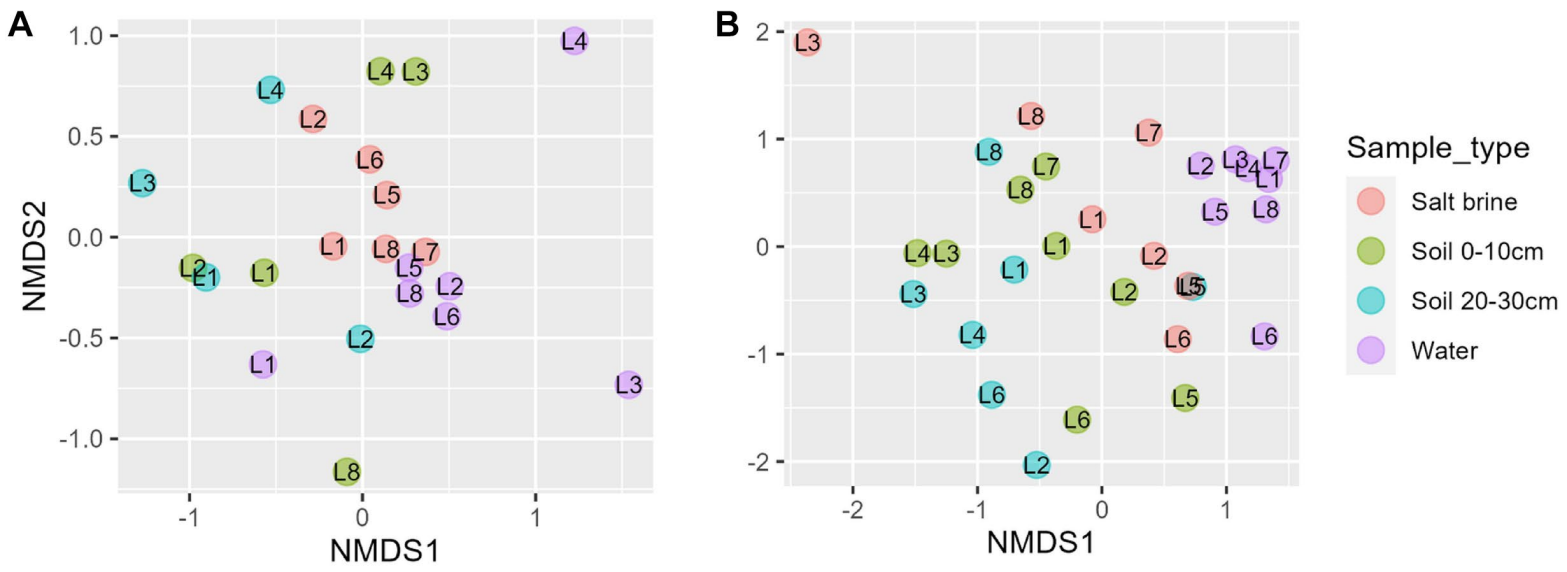
**(A)** Non-metric ordination of fungal microbial communities with a stress score of 0.18. **(B)** Non-metric ordination of eukaryotic microbial communities with a stress score of 0.20.

NMDS of the fungal dataset reveals clustering of the salt brine samples, whereas the water and soil samples had overlap (Figure 5A). Lakes 3 and 4 salt brine samples were quite distinct from the other salt brine samples. The ordination of the eukaryotic dataset shows the clustering of the water samples to the right of the ordination (Figure 5B). Soil samples at different depths had considerable overlap, and the salt brine samples did not cluster. RDA of the fungi soil and salt samples indicates an increase in sodium concentrations, which correlated with salt brine samples, with the exception of Lake 1 (Supplementary Figure 8). Ammonium, manganese, and potassium were positively correlated and associated with 0–10 cm soil samples of Lakes 3 and 4 and subsequently negatively correlated with organic carbon. ANOVA testing indicated that copper, ammonium, and organic carbon were significant drivers of the variance. RDA of the eukaryotic soil and salt samples indicates all sample types of Lake 5 and the 0–10 cm soil and salt brine samples of Lakes 2 and 6 were positively correlated with sodium and organic carbon but negatively correlated with pH, calcium, sulfur, and iron (Supplementary Figure 9). The 20–30 cm soil samples of Lakes 4 and 6 were positively correlated with conductivity, aluminum, potassium, magnesium ammonium, copper, and manganese. Increased concentrations of pH, sulfur, calcium, and iron were positively correlated with the 20–30 cm soil samples of Lake 8 and the 0–10 cm soil samples of Lake 7. ANOVA testing of the model indicates that sulfur, sodium, potassium, and ammonium were significant predictors of variance.

## 4 Discussion

In this study, we have collected data to create a snapshot of the microbial community structure of eight acidic saline lakes across the Yilgarn Craton. It is evident that these lakes harbor an extremely hostile environment for biological life; however, we still observe great microbial diversity. We aimed to highlight microbes in the lakes that may have biotechnological applications as well as what environmental conditions drive differences in the microbial communities between the lakes. The biotechnological applications of halophiles and acidophiles are well known. Halophiles are sought for their production of compatible solutes, bioactive enzymes with pharmaceutical applications, pigment production, biofuel production, and many more (Dutta and Bandopadhyay, 2022). Acidophiles have been utilized for their acid-stable enzymes in baking, fruit processing, pharmaceuticals, biofuel production, and bioleaching (Rampelotto, 2016). Saline environments that are also acidic have been studied in the past in relation to Mars analogs. Through the exploration of the microbial ecology of these lakes and highlighting the abundant microbial players, we hope to facilitate focused bioprospecting efforts for microbes with application in saline biomining operations. In order to capture microbial communities exposed to extremely acidic conditions, lake selection was limited to those with water pH below 4. All water samples had a pH between 3.7 and 3.2; however, the soil and salt brine samples were between pH 4 and 6.5; thus, the soil and salt brine microbial communities are theoretically less adapted to acidity than the water samples.

### 4.1 Environmental drivers of the microbial communities

To gain an understanding of how the microbial communities in these acidic saline lakes relate to the geochemical data collected in this study, we utilized RDA analysis. Soil and salt brine samples were combined in the same RDA analysis as they were subject to the same chemical testing. Salt brine samples correlated with increased sodium concentrations across the range of organism classifications. The bacterial RDA model explained 20% of the constrained variance. Increased sodium, carbon, and sulfur were correlated with the salt brine samples, whereas increased manganese, copper, ammonium, conductivity, potassium, magnesium, and aluminum were correlated with the soil samples. ANOSIM identified sulfur, ammonium, organic carbon, and potassium as being the most significant variables in explaining the bacterial community variance. These chemicals are important in these environments as they provide energy sources, osmotic activity, and pH regulation in bacterial communities. Organic carbon and ammonium were repeatedly identified as significant in the RDA models for all the datasets, highlighting the importance of carbon and ammonium in the microbial composition of these lakes. Interestingly, sulfur and potassium were significant in the bacterial and eukaryotic models but not in the archaeal or fungal models. Copper was significant in the archaeal and fungal models but not in the bacterial or eukaryotic models. Copper concentrations in the lakes were extremely low, ranging from 0.17 to 1.52 ppm. Copper is an essential cofactor for multiple enzymes. Across all the datasets, the redundancy analysis models only explained between 16 and 27% of the variation in the microbial community composition. This may indicate that environmental parameters not measured in this study could be important drivers of communities. It would be preferable to do a longitudinal study of the change in microbial communities and environmental parameters to gain a better understanding of the environmental drivers in these lakes.

### 4.2 Microbial communities with possible biotechnological applications

The subset of bacteria previously identified in the biotechnological application of biomining was examined closely in the context of these acidic saline lakes. The ordination of this subset of iron- and/or sulfur-oxidizing and/or reducing bacteria revealed that the iron- and sulfur-reducing bacteria were closely related to the soil samples at 20–30 cm. This is to be expected, as iron and sulfur reduction processes require anaerobic conditions. Iron- and sulfur-oxidizing bacteria were associated with both soil depths. No genera had significant positive correlations with the sulfur concentrations; however, it is important to note that there was very little variation in sulfur concentration between the lakes sampled. There was a significant positive correlation between iron concentration and the presence of *Desulfosporosinus*, *Desulfopila*, and *Acidocella*. *Desulfosporosinus* and *Desulfopila* are acidophilic sulfate and iron-reducing bacteria and are commonly found in acid mine drainage environments (Bertel et al., 2012). *Acidocella* species have the capacity to reduce iron, therefore, a positive correlation with the iron concentration is expected. Although there are some significant correlations between the environmental data and the biotechnologically important genera, it is difficult to draw any conclusions on how lake conditions influence microbial communities as there is little environmental variation between several tested parameters. While bacteria that have been utilized in biomining were identified in this study, they were not dominant in the lakes sampled. This could be an indication that these lakes, high in acidity and salinity, are, as previously mentioned, an evolutionarily challenging environment. Multiple iron- and sulfur-oxidizing bacteria not previously used in biomining have been identified in this study, possibly indicating these acidic saline lakes in WA are ideal sites to search for novel candidates in biomining.

### 4.3 Bacterial communities

Water samples were the least diverse of all sample types, with soil samples being the most diverse. The low diversity of the water samples could be due to the high abundance of contaminating chloroplast and mitochondrial reads. *Acetobacteraceae* was one of the most abundant bacterial families in the salt brine and water samples, consisting of a combination of the *Acidocella* genus, the *Acidiphilium* genus, and multiple unclassified genera. Although these were the most abundant *Acetobacteraceae* genera in the water samples, this was only seen in specific lakes. *Acidiphilium* was in high abundance only in Lake 1 (13.4%) and Lake 3 (40.5%), and *Acidocella* was only abundant in Lake 4 (59%). *Acidocella* is a mesophilic, heterotrophic, iron-reducing acidophile that currently has four species and has been identified in acid mine drainage environments. The most abundant ASV in our dataset that contributed to 99% of the *Acidocella* reads in Lake 4 had the highest identity (97.69%) to *Acidocella aminolytica* and was exclusively present in the water samples of Lakes 3 and 4. No one environmental parameter was overly similar between Lakes 3 and 4, apart from their proximity, which was approximately 300 m between the lakes. The ability of *Acidocella* to produce gold nanoparticles in the recovery of printed circuit board waste has been investigated (Intan Nurul and Okibe, 2018). The most abundant *Acidiphilium* ASV in Lakes 1 and 3 water samples had the highest identity to *A. multivorum* (97.96 and 98.41%, respectively), an acidophilic, gram-negative, chemoorganotrophic sulfur-oxidizing bacteria that was isolated from AMD in Japan (Wakao et al., 1994). This genus has been hypothesized to be used in the production of acetic acid, is metal-resistant, and has also been identified as an extremely active sulfur oxidizer in industrial bioreactors leaching gold from pyrite–arsenopyrite concentrates (Micciche et al., 2020; Panyushkina et al., 2021). *Salinisphaeraceae* was another family in high abundance in the salt brine and water samples and consisted exclusively of the *Salinisphaera* genus, which is an aerobic facultative chemolithoautotrophic halophile and has been isolated from slightly acidic environments (Crespo-Medina et al., 2009; O’Dell et al., 2018). ASV8, the most dominant *Salinisphaera* read in the Lakes 6 and 7 water samples, and ASV17, the most abundant *Salinisphaera* read in Lakes 6 and 8, had the highest identity (94.67% and 94.47%, respectively) to *S. aquimarina*. No biotechnological applications of *S. aquimarina* have been identified in the current literature. The *Acidithiobacilliaceae* family was the most dominant family in the soil samples. The *Acidithiobacilliaceae* family includes the well-known *Acidithiobacillus* genus of extreme acidophiles that are susceptible to chloride ions. However, almost all ASVs in the *Acidithiobacilliaceae* family were classified as the uncultured 9 M32 genus. This brings into question whether the 9 M32 is possibly a genus of *Acidithiobacilliaceae* that is resistant to chloride ions and may have application in saline biomining. Previous research utilizing metagenomic data identified this genus in groundwater samples of another slightly acidic saline lake in WA and was related to putative sulfide-oxidizing microbes (Zaikova et al., 2018). Further sampling of Lake 6 soil samples would be ideal to possibly isolate and culture this microbe. The *Inmirania* genus was abundant in the 0–10 cm soil samples of Lake 5 (17.7%) and had the highest identity to *I. thermothiophila* (92%), which is a thermophilic sulfur oxidizer that was isolated from a shallow thermal vent in Russia (Slobodkina et al., 2016). *I. thermothiophila* has not been associated with any biotechnological applications, but its thermotolerance and sulfur oxidation capabilities may make it a good candidate to explore.

### 4.4 Archaeal communities

*Halobacteriaceae*, the most abundant family in the archaeal dataset, almost exclusively consisted of the *Halarchaeum* genus. The 0–10 cm soil sample in Lake 2 and the salt brine samples from Lakes 1 and 5 had the highest abundance of *Halarchaeum* ASVs, whereas they were in extremely low numbers in all other samples. *Halarchaeum* currently has six identified species, a number of which are moderately acidophilic and have a range of metabolic capabilities. The most abundant *Halarchaeum* ASVs had the highest identity to *H. grantii*, which is an aerobic, moderately acidophilic haloarchaeon (optimum pH of 5.5; Shimane et al., 2015). The majority of *Halarcheum* spp. have been isolated from natural saline environments and are mostly neutrophilic or alkaliphilic; therefore, identifying *Halarcheum* spp. in these acidic saline lakes may result in the discovery of more acidophilic *Halarcheum* species (Oren, 2017). Members of the Nanohaloarchaeota phylum are symbiotes of other archaea and are characterized by their nano-sized cells (Zhao et al., 2022). Lake 1 (0–10 cm), Lake 7 (0–10 cm and 20–30 cm soil), Lake 5 (salt brine), and Lake 8 water had approximately 50% of their archaeal 16S rRNA V3-V4 reads classified as Candidatus *Haloredivivus*. These reads were absent from all sample types in Lake 2 and in low amounts in Lakes 4 and 7. Candidatus *Haloredivivus* was originally identified in a hypersaline pond in Spain, and through genome analysis, it is hypothesized to live a photoheterotrophic lifestyle (Ghai et al., 2011). The 20–30 cm soil profiles of Lake 1, Lake 6, and Lake 8 were dominated by unclassified *Nitrosotaleaceae*. Although these reads could not be classified past family level, members of the *Nitrosotaleaceae* family have been identified in AMD environments and are known for their ability to oxidize ammonia for energy and play an important role in nitrification in acidic conditions (Gupta et al., 2021). *Haloferacaceae* was the second most abundant archaeal family in the dataset and had the highest abundance in the soil samples; however, it was not present in either of the Lake 2 soil samples or the 20–30 cm samples of Lake 10. The majority of *Haloferacaceae* ASVs were not classified past the family level. In some samples, *Halorussus*, *Halolamina*, and *Halo*var*ius* were more abundant than unclassified *Haloferacaceae* reads. These genera are extremely halophilic gram-negative bacteria, are typically neutrophilic, and are aerobes (Oren and Ventosa, 2015). The archaeal communities in these lakes are dominated by extremely halophilic archaea. Although some genera identified in these lakes are moderately acidophilic, the lack of known acidophilic archaea may be an indication that salt stress is the more important environmental stressor that these communities are subjected to rather than acidity. Halophilic archaea are known to possess a multitude of functional proteins and metabolites that have applications in biotechnology (Singh and Singh, 2017).

### 4.5 Fungal communities

The fungal communities on average had the lowest diversity measures compared to the other microbial communities, with little variation in diversity between sample types. The greatest fungal diversity was observed in the 0–10 cm soil samples from Lakes 3 and 4 (4.19 and 4.09). The water sample from Lake 1 had the lowest fungal diversity (0.76). *Sporormiaceae* was an abundant family of fungi across the salt brine and 0–10 cm soil samples, which contain mostly saprobic coprophilous organisms. *Preussia persica* accounted for 40% of all *Sporormiaceae* reads and has been isolated from fecal matter and soil from across the world. This species of fungus is not known to be isolated in saline environments and has been of interest in previous studies searching for bioactive secondary metabolites (Gonzalez-menendez et al., 2017). The *Mycosphaerellaceae* family was most abundant in water samples, and almost all reads in this family were classified as *Mycosphaerella tassiana*, a phytopathogenic fungus that has been screened for amylase production (Saleem and Ebrahim, 2014). *Pleosporaceae* was made up of multiple genera in this dataset, including *Alternaria* spp., *Stemphylium solani*, and *Neocamarosporium* spp. These organisms are mostly known for their crop pathogenicity. The majority of the *Didymellaceae* family was not classified past its family level and is an incredibly species-rich family that is known to be phytopathogenic. Many of the largest fungi families identified in these lakes are not known for their association with saline or acidic environments. It is unclear if the fungi identified in these lakes were truly adapted to the saline and acidic environment or if they are dormant and merely surviving. If, in fact, the fungal species in these lakes are truly adapted to the saline and acidic conditions of the environment, further research may be able to elucidate their application in biotechnological applications, as for other halotolerant fungi such as biofuel.

### 4.6 Eukaryotic communities

The eukaryotic communities were generally less diverse compared to the bacterial and archaeal communities. The lowest average eukaryotic diversity was observed in the water samples, whereas the 0–10 cm soil samples had a range of diversities between the lakes. The majority of the eukaryotic sequences were not classified past the kingdom level, highlighting the lack of phylogenetic clarity of the eukaryotic microbes that inhabit these environments. *Dunaliella salina*, an alga commonly found in hypersaline environments, was the most abundant eukaryote in the water samples. *D. salina* has been of interest for its high carotenoid, lipid, and glycerol content, ideal for application in the cosmetic industry and biofuels (Monte et al., 2020). Common eukaryotes found in acidic environments were absent from these lakes.

## 5 Conclusion

Acid-saline lakes, although described as having some of the most biologically hostile conditions, clearly harbor a wide diversity of microorganisms. Through this study, we hoped to gain insight into the environmental conditions that drive microbial community composition and to understand what types of iron- and sulfur-oxidizing bacteria and archaea with potential applications in biomining are inhabiting these lakes. Further studies of these lakes should be conducted to isolate more novel biomining organisms, and the current study has identified that soil samples are the best area to target in this quest. A future study of these lakes will also shed light on the evolutionary mechanisms of haloacidophiles, which is an evolutionarily rare occurrence.

## Data availability statement

The datasets presented in this study can be found in online repositories. The names of the repository/repositories and accession number(s) can be found at: NCBI—PRJNA1030493.

## Author contributions

KB: Data curation, Methodology, Writing – original draft, Writing – review & editing. TS: Conceptualization, Resources, Supervision, Writing – review & editing. EW: Conceptualization, Funding acquisition, Resources, Supervision, Writing – review & editing.

## Footnotes

^1^ https://www.australianmicrobiome.com/protocols/

^2^ https://www.bioinformatics.babraham.ac.uk/projects/fastqc/
