# Supplementary material for: Microbes of biotechnological importance in acidic saline lakes in the Yilgarn Craton, Western Australia
Source: Front Microbiol. 2024 Feb 14;15:1308797. doi: 10.3389/fmicb.2024.1308797 (PMC10899397; doi:10.3389/fmicb.2024.1308797)
Supplement: Supplementary file 1 [file Data_Sheet_1.docx]

Supplementary Material

# Sample Metadata

Table 1: Information of the samples collected and their relevant chemical data.

| Sample name | Date sampled  (YYYY-MM-DD) | Latitude | Longitude | Sample type | Conductivity (dS/m) | Nitrate (mg/Kg) | Ammonium (mg/Kg) | P (mg/Kg) | K (mg/Kg) | S (mg/Kg) | Organic Carbon (%) | pH Solid CaCl2 | pH Solid H2O | Cu (mg/Kg) | Fe (mg/Kg) | Mn (mg/Kg) | Zn (mg/Kg) | Al (meq/100g) | Ca (meq/100g) | Mg (meq/100g) | K(meq/100g) | Na (meq/100g) | Boron (mg/Kg) |
| --- | --- | --- | --- | --- | --- | --- | --- | --- | --- | --- | --- | --- | --- | --- | --- | --- | --- | --- | --- | --- | --- | --- | --- |
| L1_0-10cm | 2020 06 08 | 117.86987 | -31.217296 | Soil | 4.702 | 1 | 11 | <2 | 364 | 1944.6 | 1.25 | 4.5 | 4.7 | 0.46 | 90.7 | 1.79 | 0.28 | 0.22 | 1.66 | 6.6 | 0.58 | 49.43 | 4.51 |
| L2_0-10cm | 2020 06 08 | 117.83174 | -31.190272 | Soil | 2.849 | 2 | 3 | <2 | 473 | 407.2 | 0.95 | 4.1 | 4.2 | 0.22 | 57.6 | 0.41 | 0.27 | 0.77 | 0.26 | 4.24 | 0.66 | 18.33 | 5.56 |
| L3_0-10cm | 2020 06 09 | 118.76272 | -31.008657 | Soil | 6.329 | 1 | 8 | 2 | 473 | 678 | 0.74 | 4.6 | 4.7 | 0.52 | 47.9 | 13.76 | 0.3 | 0.37 | 0.72 | 11.36 | 0.97 | 37.35 | 8.42 |
| L4_0-10cm | 2020 06 09 | 118.76588 | -31.010387 | Soil | 9.572 | 2 | 36 | 10 | 1220 | 1255.5 | 1.03 | 6.5 | 6.5 | 1.52 | 71.9 | 63.69 | 0.9 | 0.05 | 3.84 | 40.64 | 2.63 | 133.85 | 9.1 |
| L5_0-10cm | 2020 06 10 | 117.40826 | -31.01137 | Soil | 10.997 | <1 | 8 | <2 | 480 | 507.7 | 0.97 | 4.2 | 4.3 | 0.43 | 61.1 | 1.5 | 0.23 | 0.4 | 1 | 6.23 | 1.06 | 37.4 | 9.19 |
| L6_0-10cm | 2020 06 10 | 117.40462 | -31.007898 | Soil | 5.14 | 9 | 7 | <2 | 619 | 372.5 | 0.93 | 4.2 | 4.3 | 0.72 | 30.8 | 0.72 | 0.16 | 0.64 | 0.39 | 6.93 | 1.04 | 24.15 | 7.2 |
| L7_0-10cm | 2020 06 10 | 117.33024 | -31.049602 | Soil | 5.978 | <1 | 1 | <2 | 392 | 10061.2 | 0.38 | 6.1 | 6.3 | 0.43 | 17.8 | 2.11 | 0.22 | 0.06 | 15.23 | 5.06 | 0.8 | 21.55 | 3.95 |
| L8_0-10cm | 2020 06 10 | 117.10198 | -31.04831 | Soil | 2.658 | <1 | 1 | 2 | 85 | 93.7 | 0.21 | 4.6 | 5.1 | 0.2 | 85.3 | 0.45 | 0.13 | 0.07 | 0.22 | 1.06 | 0.15 | 12.06 | 0.91 |
| L1_20-30cm | 2020 06 08 | 117.86987 | -31.217296 | Soil | 10.752 | 1 | 5 | <2 | 324 | 253 | 0.53 | 4.4 | 4.7 | 0.48 | 45.9 | 0.53 | 0.28 | 0.34 | 0.68 | 3.3 | 0.64 | 18.36 | 2.56 |
| L2_20-30cm | 2020 06 08 | 117.83174 | -31.190272 | Soil | 58.944 | <1 | 2 | <2 | 256 | 163.7 | 0.37 | 4.2 | 4.3 | 0.27 | 53.6 | 0.27 | 0.32 | 0.53 | 0.28 | 1.77 | 0.42 | 9.82 | 3.36 |
| L3_20-30cm | 2020 06 09 | 118.76272 | -31.008657 | Soil | 51.924 | <1 | 6 | <2 | 410 | 284.6 | 0.5 | 4.4 | 4.6 | 0.95 | 65.3 | 7.18 | 0.54 | 0.33 | 0.61 | 6.14 | 0.85 | 27.11 | 4.61 |
| L4_20-30cm | 2020 06 09 | 118.76588 | -31.010387 | Soil | 188.388 | 1 | 10 | 3 | 565 | 428.1 | 0.46 | 4.2 | 4.2 | 0.79 | 51.9 | 6.3 | 0.3 | 0.73 | 1.38 | 9.69 | 1.26 | 41.26 | 4.99 |
| L5_20-30cm | 2020 06 10 | 117.40826 | -31.01137 | Soil | 113.836 | <1 | 10 | 14 | 654 | 1023.3 | 0.82 | 4.1 | 4.2 | 0.97 | 67.9 | 2.16 | 0.55 | 0.72 | 0.94 | 9.68 | 1.35 | 50.59 | 10.9 |
| L6_20-30cm | 2020 06 10 | 117.40462 | -31.007898 | Soil | 32.04 | 7 | 5 | <2 | 584 | 307.3 | 0.3 | 4.1 | 4.2 | 0.47 | 7.8 | 0.48 | 0.14 | 0.75 | 0.46 | 5.13 | 1.16 | 20 | 4.55 |
| L8_20-30cm | 2020 06 10 | 117.10198 | -31.04831 | Soil | 28.94 | <1 | <1 | <2 | 67 | 88.6 | 0.1 | 4.5 | 4.8 | 0.3 | 60.9 | 0.32 | 0.29 | 0.09 | 0.11 | 0.82 | 0.11 | 9.99 | 0.77 |
| L1_SB | 2020 06 08 | 117.86986 | -31.217211 | Salt brine | 10.825 | 1 | 11 | <2 | 466 | 1737.4 | 1.71 | 4.5 | 4.7 | 0.27 | 66.8 | 2.09 | 0.3 | 0.37 | 2.1 | 7.67 | 0.82 | 56.37 | 3.77 |
| L2_SB | 2020 06 08 | 117.83143 | -31.190347 | Salt brine | 5.776 | 1 | 11 | <2 | 852 | 2760.6 | 0.84 | 4.5 | 4.5 | 0.17 | 142.1 | 2.02 | 0.4 | 2.36 | 1.7 | 25.55 | 1.29 | 228.09 | 13.81 |
| L3_SB | 2020 06 09 | 118.76268 | -31.008767 | Salt brine | 8.934 | 3 | 21 | 4 | 1155 | 9708.1 | 1.3 | 4.4 | 4.5 | 0.62 | 152.3 | 13.86 | 0.5 | 3.04 | 7.34 | 45.84 | 2.64 | 257.07 | 26.53 |
| L5_SB | 2020 06 10 | 117.40809 | -31.011601 | Salt brine | 8.761 | <1 | 8 | <2 | 412 | 1710 | 1.15 | 5.4 | 5.5 | 0.56 | 18.1 | 19.46 | 0.96 | 0.1 | 1.44 | 20.24 | 0.8 | 528.27 | 3.76 |
| L6_SB | 2020 06 10 | 117.40468 | -31.008093 | Salt brine | 6.611 | 24 | 10 | <2 | 614 | 1981.9 | 0.8 | 4.8 | 4.9 | 0.21 | 15.2 | 2.89 | 0.22 | 0.65 | 0.83 | 33.63 | 1.25 | 190.14 | 7.79 |
| L7_SB | 2020 06 10 | 117.33036 | -31.049483 | Salt brine | 7.344 | <1 | 1 | <2 | 48 | 8499.3 | 0.12 | 5.9 | 6.2 | 0.27 | 4.9 | 0.36 | 0.24 | 0.02 | 8.09 | 1.49 | 0.07 | 6.69 | 0.9 |
| L8_SB | 2020 06 10 | 117.10219 | -31.048304 | Salt brine | 3.812 | <1 | 3 | 3 | 212 | 642.3 | 0.91 | 4.9 | 5 | 0.24 | 124.8 | 3.26 | 0.25 | 0.14 | 0.85 | 4.88 | 0.44 | 129.4 | 3.04 |
| Sample name | Date sampled (YYYY-MM-DD) | Latitude | Longitude | Sample type | Conductivity (dS/m) | Nitrate Nitrogen (mg/Kg) | pH Aqueous (pH) | P (mg/L) | S (mg/L) | Ch (mg/L) | Carbonate /Bicarbonate (mg/L) | Cu (mg/L) | Fe (mg/L) | Mn (mg/L) | Zn (mg/L) | Ca (mg/L) | Mg (mg/L) | K (mg/L) | Na (mg/L) | B (mg/L) |  |  |  |
| WL1 | 2020 06 08 | 117.86983 | -31.217234 | Water | 96.914 | <0.1 | 3.6 | <0.05 | 1996 | 40500 | 0 | <0.05 | 1.41 | 2.2 | 0.07 | 1690 | 2085 | 335 | 29310 | 6.02 |  |  |  |
| WL2 | 2020 06 08 | 117.83148 | -31.190064 | Water | 131.513 | 0.1 | 3.3 | <0.05 | 2420 | 63000 | 0 | <0.05 | 7.43 | 1.77 | 0.07 | 1634 | 3155 | 602.7 | 42380 | 15.72 |  |  |  |
| WL3 | 2020 06 09 | 118.76326 | -31.008502 | Water | 119.312 | 2.35 | 3.2 | <0.05 | 1539 | 54500 | 0 | <0.05 | 4.87 | 5.22 | <0.05 | 735.1 | 3635 | 622.9 | 34280 | 13.22 |  |  |  |
| WL4 | 2020 06 09 | 118.76584 | -31.010976 | Water | 79.808 | 3.45 | 3.4 | <0.05 | 901.4 | 34000 | 0 | <0.05 | 3.78 | 2.19 | <0.05 | 421 | 2142 | 365.5 | 21330 | 7.51 |  |  |  |
| WL5 | 2020 06 10 | 117.4088 | -31.011206 | Water | 136.038 | <0.1 | 3.5 | <0.05 | 2483 | 66000 | 0 | <0.05 | 1.75 | 4.45 | 0.19 | 1931 | 3736 | 928.9 | 41790 | 11.9 |  |  |  |
| WL6 | 2020 06 10 | 117.404 | -31.007884 | Water | 216.701 | 18.74 | 3.4 | <0.05 | 1977 | 17800 | 0 | <0.05 | 1.44 | 5.13 | <0.05 | 1145 | 3099 | 818.1 | 116500 | 7.52 |  |  |  |
| WL7 | 2020 06 10 | 117.3301 | -31.049175 | Water | 67.734 | <0.1 | 3.7 | <0.05 | 1814 | 28000 | 0 | <0.05 | 3.75 | 1.54 | <0.05 | 1517 | 1475 | 374.7 | 18030 | 3.5 |  |  |  |
| WL8 | 2020 06 10 | 117.10197 | -31.047738 | Water | 167.911 | 0.13 | 3.5 | <0.05 | 1035 | 93500 | 0 | <0.05 | 12.07 | 3.34 | <0.05 | 610.6 | 1137 | 323.4 | 61770 | 3.79 |  |  |  |

# Supplementary Methods: Amplicon Data Processing

All filtering, trimming, merging and chimera removal for all sequences was done using DADA2 following the recommendations provided in the DADA2 16S and ITS amplicon processing tutorial (<https://benjjneb.github.io/dada2/tutorial.html> and <https://benjjneb.github.io/dada2/ITS_workflow.html>). The Silva 16S non-redundant 99 trainset v138.1 was used for taxonomic assignment of the 16S and A16S sequences, UNITE dynamic release (10^th^ May 2021) was used for the ITS sequences and the Silva 18S non-redundant 99 trainset was used for 18S sequences. The package Microdecon was used to remove contaminant sequences from the soil and salt brine samples with the blank milk samples used as blanks. The bacterial 16S, A16S, ITS and 18S datasets were rarefied to 8200, 8200, 1700 and 8500 reads per sample respectively. This resulted in the loss of 7 samples from the ITS due to insufficient sequence numbers in these samples. Addition processing information can be viewed in Table 2.

Table 2: Read counts of the amplicon sequences through the steps of processing with DADA2.

|  | **Sample** | **Input** | **Filtered** | **DenoisedF** | **DenoisedR** | **Merged** | **Chimeras removed** | **Post Decon-tamination** | **Rarefied** |
| --- | --- | --- | --- | --- | --- | --- | --- | --- | --- |
| 16S rRNA V1-V3 | S_0-10_L1 | 77218 | 52453 | 36938 | 37462 | 31198 | 29996 | 29708 | 8200 |
|  | S_0-10_L2 | 82636 | 33665 | 24589 | 24662 | 24183 | 23967 | 22548 | 8200 |
|  | S_0-10_L3 | 74884 | 62464 | 41954 | 44536 | 28898 | 25808 | 25774 | 8200 |
|  | S_0-10_L4 | 90648 | 70954 | 45974 | 48877 | 32516 | 29472 | 29420 | 8200 |
|  | S_0-10_L5 | 89584 | 50565 | 33835 | 33796 | 32336 | 30671 | 29092 | 8200 |
|  | S_0-10_L6 | 92022 | 48273 | 33986 | 34446 | 33465 | 32955 | 29694 | 8200 |
|  | S_0-10_L7 | 72294 | 43001 | 26990 | 27514 | 20859 | 19829 | 19696 | 8200 |
|  | S_0-10_L8 | 123881 | 81255 | 55582 | 56304 | 48205 | 46440 | 45641 | 8200 |
|  | S_20-30_L1 | 77320 | 48937 | 30869 | 30888 | 26325 | 25586 | 25380 | 8200 |
|  | S_20-30_L2 | 43679 | 28322 | 19735 | 19759 | 15373 | 15174 | 13593 | 8200 |
|  | S_20-30_L3 | 85898 | 54848 | 33262 | 34159 | 26557 | 25487 | 25168 | 8200 |
|  | S_20-30_L4 | 63133 | 39157 | 25255 | 25846 | 21331 | 20914 | 20586 | 8200 |
|  | S_20-30_L5 | 60798 | 42794 | 30465 | 30660 | 28413 | 27719 | 27206 | 8200 |
|  | S_20-30_L6 | 56507 | 29933 | 20213 | 20264 | 18834 | 18346 | 16382 | 8200 |
|  | S_20-30_L7 | 107849 | 49446 | 34737 | 34839 | 31740 | 31396 | 29593 | 8200 |
|  | S_SB_L1 | 77812 | 54890 | 42621 | 42988 | 38903 | 37709 | 37211 | 8200 |
|  | S_SB_L2 | 53725 | 33310 | 26946 | 27173 | 25767 | 25213 | 19592 | 8200 |
|  | S_SB_L3 | 44307 | 13663 | 11457 | 11273 | 10823 | 10740 | 8253 | 8200 |
|  | S_SB_L5 | 61960 | 47240 | 35745 | 36087 | 33545 | 30787 | 27769 | 8200 |
|  | S_SB_L6 | 56432 | 34628 | 30279 | 30271 | 29433 | 29218 | 11572 | 8200 |
|  | S_SB_L7 | 70627 | 42869 | 35127 | 34828 | 33326 | 32319 | 25159 | 8200 |
|  | S_SB_L8 | 111032 | 83116 | 65212 | 65767 | 60701 | 57558 | 55140 | 8200 |
|  | WL1 | 68314 | 53997 | 42621 | 42643 | 38400 | 36561 | 36561 | 8200 |
|  | WL2 | 76908 | 51367 | 42096 | 42295 | 40240 | 39167 | 39167 | 8200 |
|  | WL3 | 124426 | 68431 | 56388 | 56924 | 52833 | 50693 | 50693 | 8200 |
|  | WL4 | 76491 | 61976 | 52271 | 52718 | 48458 | 45926 | 45926 | 8200 |
|  | WL5 | 92340 | 79550 | 60766 | 60836 | 58129 | 53759 | 53759 | 8200 |
|  | WL6 | 76294 | 53462 | 39026 | 39449 | 36689 | 34083 | 34083 | 8200 |
|  | WL7 | 182538 | 162734 | 136707 | 136856 | 131301 | 102989 | 102989 | 8200 |
|  | WL8 | 64235 | 47792 | 37279 | 37442 | 35623 | 31560 | 31560 | 8200 |
|  | Blank1 | 73909 | 34621 | 31737 | 31645 | 31318 | 31203 | Removed | Removed |
|  | Blank2 | 44625 | 27571 | 24909 | 24909 | 24394 | 24025 | Removed | Removed |
|  | Blank3 | 139722 | 72458 | 65960 | 66079 | 65469 | 65116 | Removed | Removed |
| A16S rRNA V1-V3 | S_0-10_L1 | 180287 | 108481 | 105244 | 105171 | 85650 | 42409 | 42223 | 8200 |
|  | S_0-10_L2 | 359888 | 219626 | 217748 | 217227 | 192346 | 183840 | 182010 | 8200 |
|  | S_0-10_L3 | 89739 | 59473 | 55000 | 57406 | 38209 | 17991 | 17916 | 8200 |
|  | S_0-10_L4 | 212508 | 140964 | 136910 | 138374 | 112714 | 49332 | 48929 | 8200 |
|  | S_0-10_L5 | 222773 | 128926 | 127614 | 127132 | 120045 | 78472 | 77791 | 8200 |
|  | S_0-10_L6 | 383833 | 221438 | 219602 | 218822 | 179281 | 131364 | 128933 | 8200 |
|  | S_0-10_L7 | 236670 | 152116 | 148754 | 149118 | 132224 | 83054 | 82357 | 8200 |
|  | S_0-10_L8 | 207050 | 116634 | 114585 | 114403 | 106146 | 60658 | 60370 | 8200 |
|  | S_20-30_L1 | 131408 | 65047 | 63095 | 62624 | 59228 | 42467 | 42388 | 8200 |
|  | S_20-30_L2 | 226678 | 132143 | 130787 | 130760 | 124319 | 80630 | 80089 | 8200 |
|  | S_20-30_L3 | 153352 | 91892 | 88998 | 89224 | 77738 | 47993 | 47748 | 8200 |
|  | S_20-30_L4 | 172984 | 113044 | 111769 | 111710 | 101996 | 67782 | 67649 | 8200 |
|  | S_20-30_L5 | 189901 | 115198 | 114478 | 114256 | 105176 | 60077 | 59813 | 8200 |
|  | S_20-30_L6 | 123464 | 72789 | 72035 | 71816 | 70029 | 56189 | 54341 | 8200 |
|  | S_20-30_L7 | 85399 | 39416 | 37885 | 37418 | 35630 | 30293 | 30133 | 8200 |
|  | S_SB_L1 | 179394 | 102833 | 100655 | 100324 | 94467 | 62141 | 61441 | 8200 |
|  | S_SB_L2 | 206345 | 79133 | 77685 | 76949 | 53387 | 45421 | 37419 | 8200 |
|  | S_SB_L3 | 18485 | 11733 | 11721 | 11727 | 11715 | 10925 | 10925 | 8200 |
|  | S_SB_L5 | 237550 | 150595 | 150373 | 150385 | 148925 | 125834 | 125020 | 8200 |
|  | S_SB_L6 | 205019 | 101118 | 100127 | 99541 | 74824 | 61750 | 43469 | 8200 |
|  | S_SB_L7 | 152060 | 78689 | 77825 | 77327 | 70188 | 55299 | 49342 | 8200 |
|  | S_SB_L8 | 168582 | 76138 | 73402 | 72698 | 66322 | 49670 | 49382 | 8200 |
|  | WL1 | 256656 | 155457 | 154918 | 154655 | 145663 | 109014 | 109014 | 8200 |
|  | WL2 | 216650 | 73380 | 71595 | 70289 | 36001 | 25897 | 25897 | 8200 |
|  | WL3 | 132251 | 32817 | 31657 | 31251 | 23960 | 18272 | 18272 | 8200 |
|  | WL4 | 174965 | 53417 | 52538 | 52244 | 41910 | 32913 | 32913 | 8200 |
|  | WL5 | 290958 | 153369 | 153046 | 152683 | 149653 | 120570 | 120570 | 8200 |
|  | WL6 | 223321 | 140113 | 139763 | 139566 | 134726 | 86738 | 86738 | 8200 |
|  | WL7 | 189268 | 112968 | 111217 | 110823 | 91691 | 59703 | 59703 | 8200 |
|  | WL8 | 220835 | 144580 | 143191 | 143140 | 128058 | 55099 | 55099 | 8200 |
|  | Blank1 | 71142 | 33033 | 32733 | 32458 | 26061 | 20956 | Removed | Removed |
|  | Blank2 | 305447 | 169414 | 168859 | 168406 | 139749 | 126577 | Removed | Removed |
|  | Blank3 | 238190 | 137562 | 137075 | 136599 | 121228 | 86992 | Removed | Removed |
| ITS | S_0-10_L1 | 130824 | 68600 | 68423 | 68397 | 24785 | 24609 | 24485 | 1700 |
|  | S_0-10_L2 | 161657 | 54856 | 54566 | 54661 | 8586 | 8586 | 2011 | 1700 |
|  | S_0-10_L3 | 123500 | 78830 | 78530 | 78269 | 27280 | 26334 | 26295 | 1700 |
|  | S_0-10_L4 | 142041 | 88947 | 88703 | 88568 | 25113 | 24357 | 24113 | 1700 |
|  | S_0-10_L5 | 11712 | 6157 | 6045 | 6048 | 483 | 483 | 475 | Removed |
|  | S_0-10_L6 | 7088 | 2778 | 2718 | 2676 | 165 | 165 | 165 | Removed |
|  | S_0-10_L7 | 126056 | 69502 | 69396 | 69402 | 23018 | 23015 | 23009 | 1700 |
|  | S_0-10_L8 | 106126 | 59858 | 59666 | 59584 | 37158 | 36615 | 36444 | 1700 |
|  | S_20-30_L1 | 308784 | 165662 | 165287 | 165217 | 89769 | 89432 | 88408 | 1700 |
|  | S_20-30_L2 | 43482 | 17512 | 17466 | 17472 | 3653 | 3486 | 1719 | 1700 |
|  | S_20-30_L3 | 305000 | 166632 | 166481 | 166313 | 69878 | 64488 | 61699 | 1700 |
|  | S_20-30_L4 | 285288 | 141929 | 141584 | 141791 | 13756 | 13296 | 12361 | 1700 |
|  | S_20-30_L5 | 258509 | 118244 | 118173 | 118085 | 484 | 484 | 484 | Removed |
|  | S_20-30_L6 | 1752 | 741 | 698 | 683 | 475 | 475 | 116 | Removed |
|  | S_20-30_L7 | 418 | 156 | 127 | 127 | 103 | 103 | 103 | Removed |
|  | S_SB_L1 | 254789 | 146060 | 145435 | 145474 | 58476 | 58111 | 58011 | 1700 |
|  | S_SB_L2 | 283984 | 173225 | 172776 | 173051 | 39602 | 39481 | 38256 | 1700 |
|  | S_SB_L3 | 8258 | 3514 | 3404 | 3337 | 880 | 857 | 798 | Removed |
|  | S_SB_L5 | 195691 | 90857 | 90598 | 90495 | 11467 | 11439 | 11171 | 1700 |
|  | S_SB_L6 | 740230 | 480535 | 480260 | 479299 | 422343 | 417975 | 392690 | 1700 |
| ITS | S_SB_L7 | 789597 | 562166 | 560963 | 561665 | 22192 | 22011 | 21908 | 1700 |
|  | S_SB_L8 | 395113 | 251691 | 251245 | 251317 | 30649 | 30542 | 30361 | 1700 |
|  | WL1 | 136178 | 97932 | 97791 | 97759 | 63737 | 63548 | 63548 | 1700 |
|  | WL2 | 520374 | 346767 | 346392 | 346307 | 247870 | 246596 | 246596 | 1700 |
|  | WL3 | 360579 | 265567 | 265266 | 265123 | 9062 | 9053 | 9053 | 1700 |
|  | WL4 | 469611 | 323282 | 323093 | 323034 | 14367 | 13882 | 13882 | 1700 |
|  | WL5 | 92295 | 55579 | 55382 | 55293 | 26074 | 25528 | 25528 | 1700 |
|  | WL6 | 457699 | 302054 | 301632 | 301643 | 109969 | 108039 | 108039 | 1700 |
|  | WL7 | 107872 | 77537 | 77405 | 77384 | 585 | 572 | 572 | Removed |
|  | WL8 | 67586 | 31568 | 31500 | 31466 | 9866 | 9760 | 9760 | 1700 |
|  | Blank1 | 198 | 20 | 4 | 8 | 4 | 4 | Removed | Removed |
|  | Blank2 | 74615 | 40765 | 40580 | 40584 | 39522 | 39397 | Removed | Removed |
|  | Blank3 | 2708 | 1663 | 1651 | 1652 | 1649 | 1646 | Removed | Removed |
| 18S | S_0-10_L1 | 87349 | 85612 | 85007 | 84860 | 81802 | 81401 | 75167 | 8500 |
|  | S_0-10_L2 | 134989 | 132587 | 132302 | 132210 | 131264 | 129513 | 125273 | 8500 |
|  | S_0-10_L3 | 127496 | 124866 | 123183 | 123028 | 115185 | 113852 | 113664 | 8500 |
|  | S_0-10_L4 | 107987 | 105674 | 104119 | 104124 | 96194 | 95494 | 95078 | 8500 |
|  | S_0-10_L5 | 101053 | 98785 | 98599 | 98641 | 97617 | 84901 | 81021 | 8500 |
|  | S_0-10_L6 | 75176 | 73298 | 73199 | 73173 | 72903 | 61082 | 56640 | 8500 |
|  | S_0-10_L7 | 70351 | 67707 | 66572 | 67333 | 63476 | 63003 | 62693 | 8500 |
|  | S_0-10_L8 | 10002 | 9720 | 9413 | 9366 | 8614 | 8614 | 8589 | 8500 |
|  | S_20-30_L1 | 120263 | 117747 | 117248 | 117194 | 113411 | 111969 | 110649 | 8500 |
|  | S_20-30_L2 | 126528 | 123095 | 122935 | 122878 | 121686 | 117985 | 116035 | 8500 |
|  | S_20-30_L3 | 134562 | 131531 | 130965 | 130845 | 126747 | 125183 | 123473 | 8500 |
|  | S_20-30_L4 | 100953 | 97989 | 97630 | 97092 | 90237 | 84090 | 83454 | 8500 |
|  | S_20-30_L5 | 129302 | 127252 | 126959 | 126931 | 124907 | 122859 | 122233 | 8500 |
|  | S_20-30_L6 | 119935 | 116648 | 116326 | 116468 | 113438 | 111834 | 110636 | 8500 |
|  | S_20-30_L7 | 34415 | 33130 | 32902 | 32963 | 31970 | 31767 | 31221 | 8500 |
|  | S_SB_L1 | 120134 | 118149 | 117642 | 117253 | 115425 | 112938 | 110606 | 8500 |
|  | S_SB_L2 | 146557 | 142871 | 142395 | 142047 | 139949 | 139043 | 128641 | 8500 |
|  | S_SB_L3 | 59678 | 57831 | 57607 | 57537 | 57000 | 56384 | 51032 | 8500 |
|  | S_SB_L5 | 131130 | 128605 | 128199 | 128056 | 126896 | 125908 | 124549 | 8500 |
|  | S_SB_L6 | 87583 | 85740 | 85510 | 85525 | 85026 | 84431 | 78617 | 8500 |
|  | S_SB_L7 | 77064 | 74403 | 73686 | 74182 | 72459 | 70598 | 68138 | 8500 |
|  | S_SB_L8 | 61796 | 59493 | 58908 | 58851 | 55215 | 54911 | 54685 | 8500 |
|  | WL1 | 174298 | 171339 | 170814 | 170729 | 169623 | 167743 | 167743 | 8500 |
|  | WL2 | 151737 | 148192 | 147906 | 147487 | 146670 | 146302 | 146302 | 8500 |
|  | WL3 | 166746 | 162624 | 162172 | 161883 | 160016 | 159584 | 159584 | 8500 |
|  | WL4 | 121403 | 117722 | 117287 | 117426 | 115369 | 115077 | 115077 | 8500 |
|  | WL5 | 131356 | 129185 | 128818 | 128641 | 127833 | 125589 | 125589 | 8500 |
|  | WL6 | 104315 | 102029 | 101464 | 101875 | 100617 | 100106 | 100106 | 8500 |
|  | WL7 | 28780 | 27382 | 26918 | 27228 | 26238 | 26089 | 26089 | 8500 |
|  | WL8 | 146757 | 143309 | 142947 | 142969 | 141458 | 140374 | 140374 | 8500 |
|  | Blank1 | 63547 | 59214 | 58817 | 58491 | 58012 | 58012 | Removed | Removed |
|  | Blank2 | 75590 | 70351 | 70034 | 70200 | 68841 | 68841 | Removed | Removed |
|  | Blank3 | 34762 | 32271 | 32065 | 32144 | 31455 | 30906 | Removed | Removed |


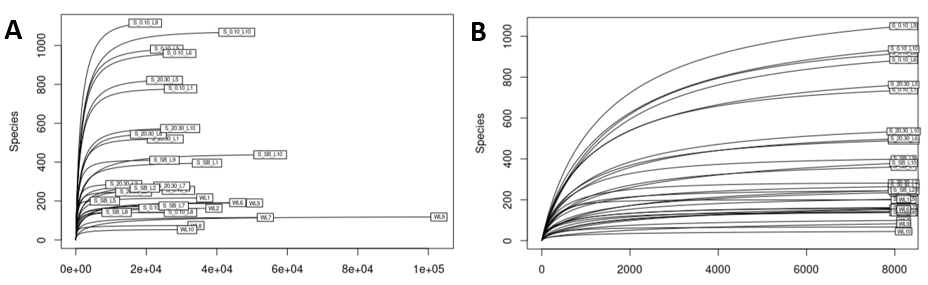


**Figure 1:** A) Bacterial dataset pre-rarefication. B) Bacterial dataset post-rarefication.


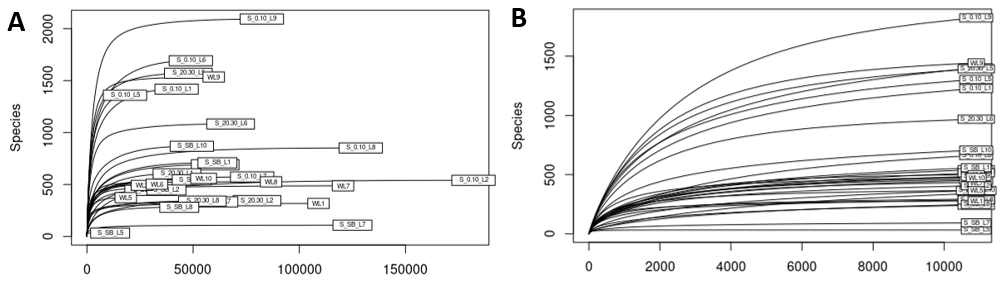


**Figure 2**: A) Archaeal dataset pre-rarefication. B) Archaeal dataset post-rarefication.


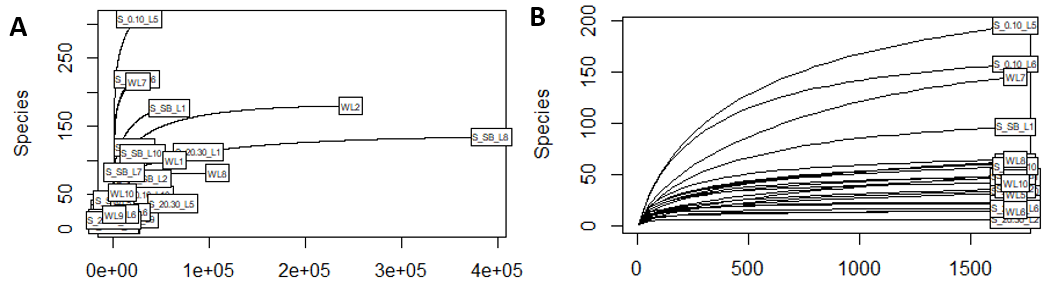


Figure 3: A) Fungal dataset pre-rarefication. B) Fungal dataset post-rarefication.


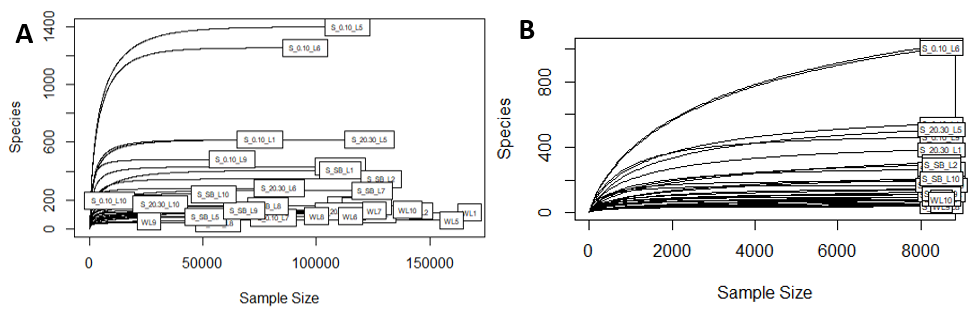


Figure 4: A) Eukaryotic dataset pre-rarefication. B) Eukaryotic dataset post-rarefication.

# Additional analysis


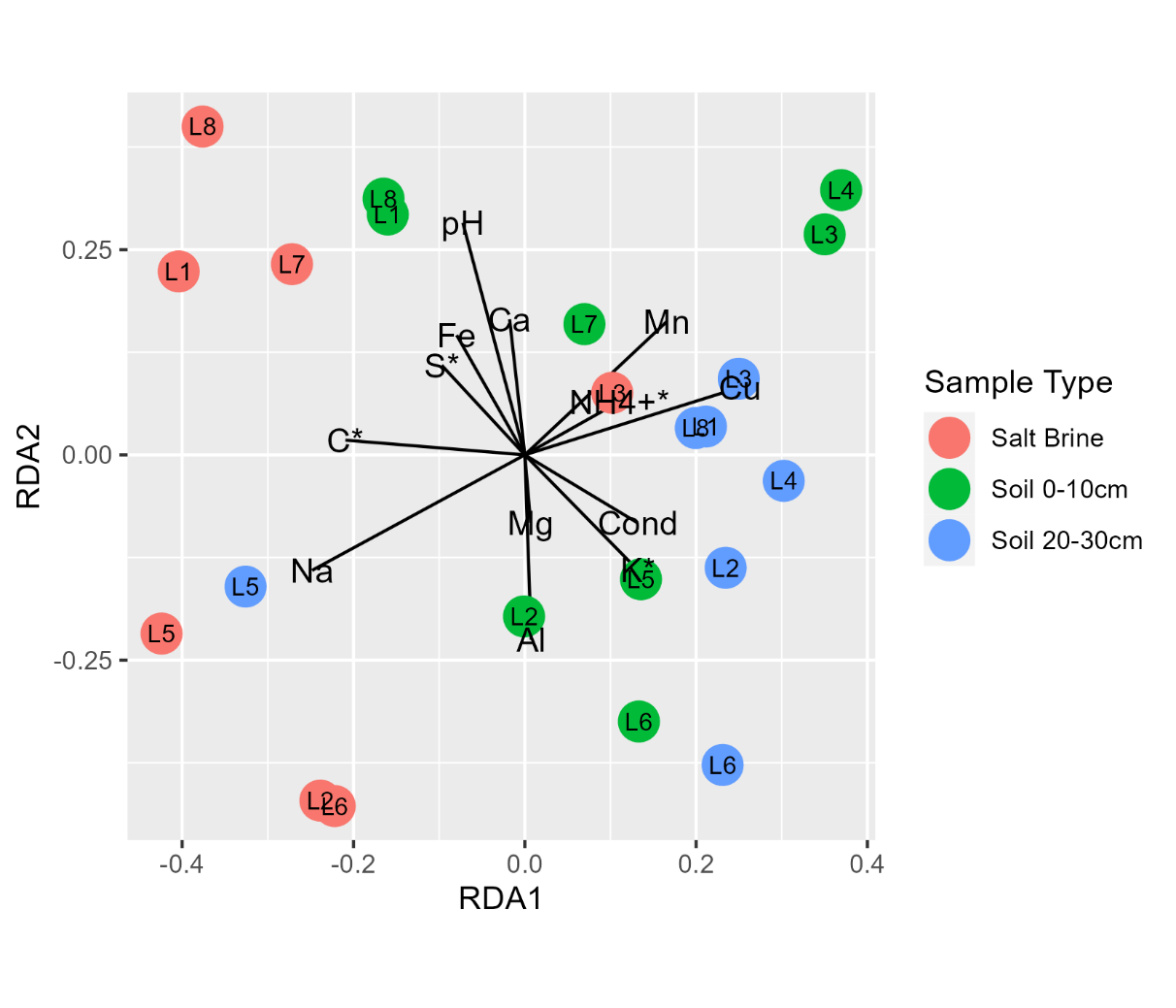


Figure 5: RDA analysis of the Bacterial populations in soil and salt samples. Proportion explained, RDA1 10.6%, RDA2 9%. Adjusted R squared = 0.12. ANOVA testing of the model had a p value of 0.009. Environmental data that was significant in explaining variance by ANOVA is indicated by an asterisk.


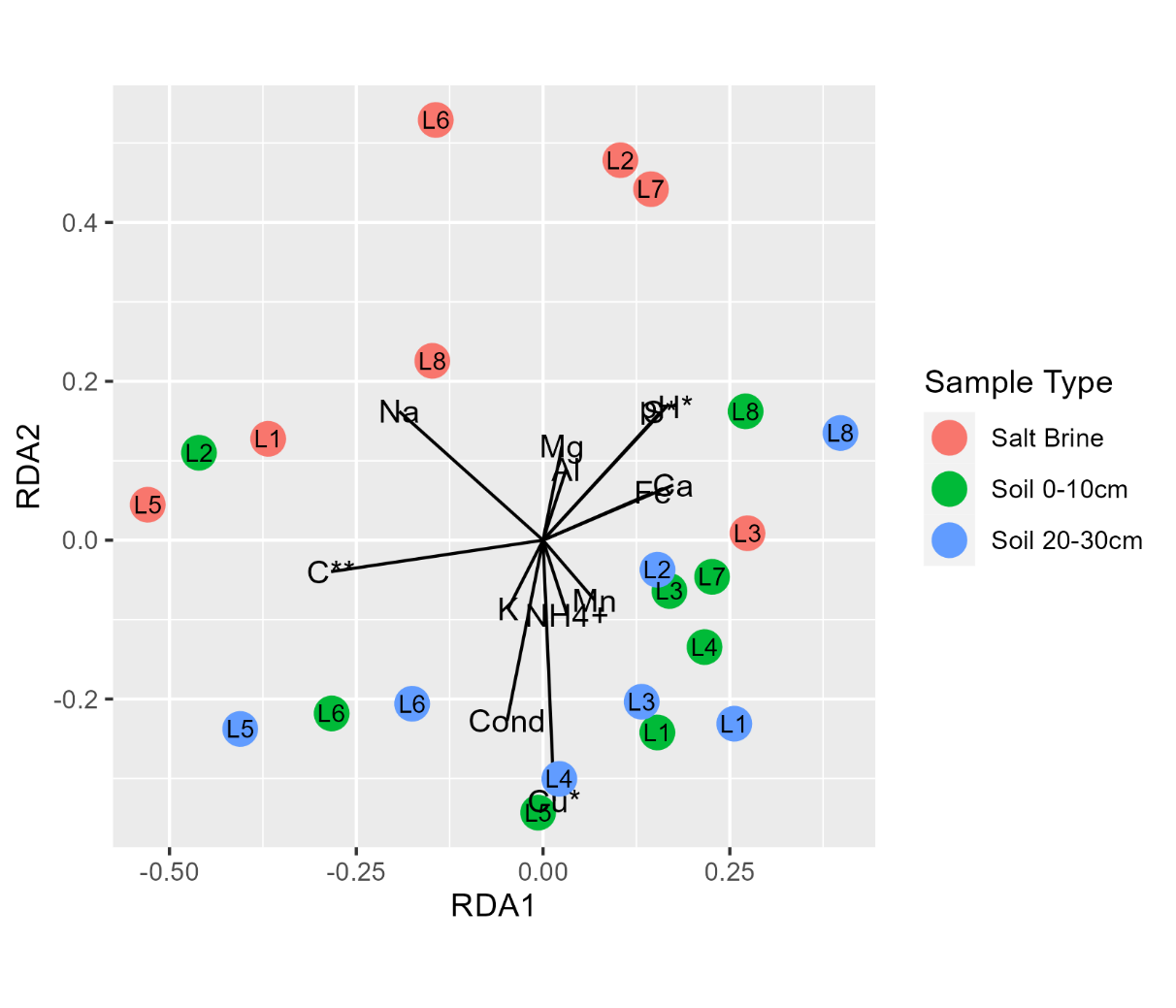


Figure 6: RDA analysis of the Archaeal populations in soil and salt samples. Proportion explained, RDA1 11.6%, RDA2 9.3%. Adjusted R squared = 0.12. ANOVA testing of the model had a p value of 0.009. Environmental data that was significant in explaining variance by ANOVA is indicated by an asterisk.


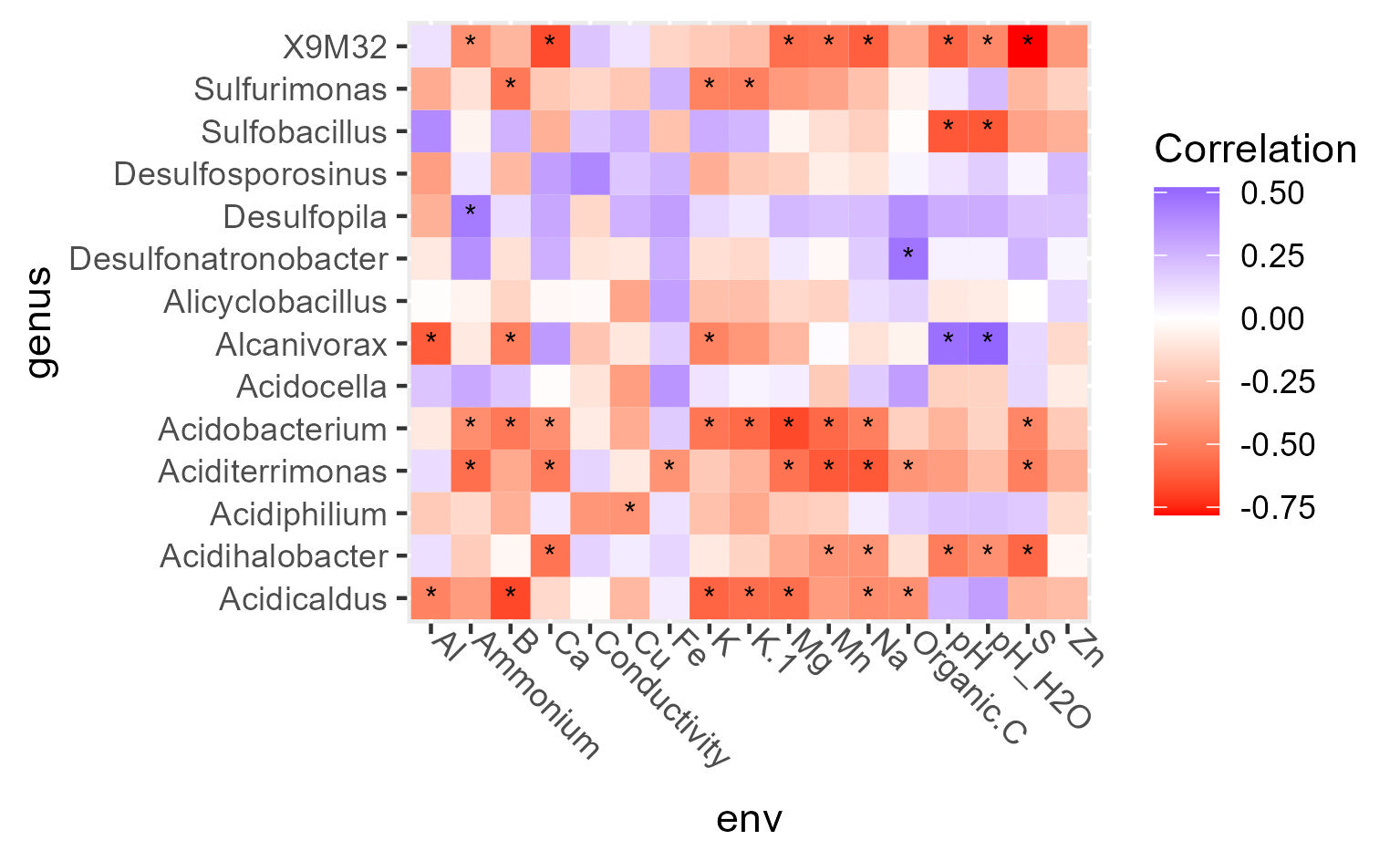


Figure 7: Spearman correlation of the subset of possible iron and/or sulfur oxidizing/reducing bacteria in the soil and salt brine samples Hellinger transformed abundance table against standardized chemical data using Hmisc R package rcorr Significant correlations with p values below 0.05 are indicated with an asterisk.


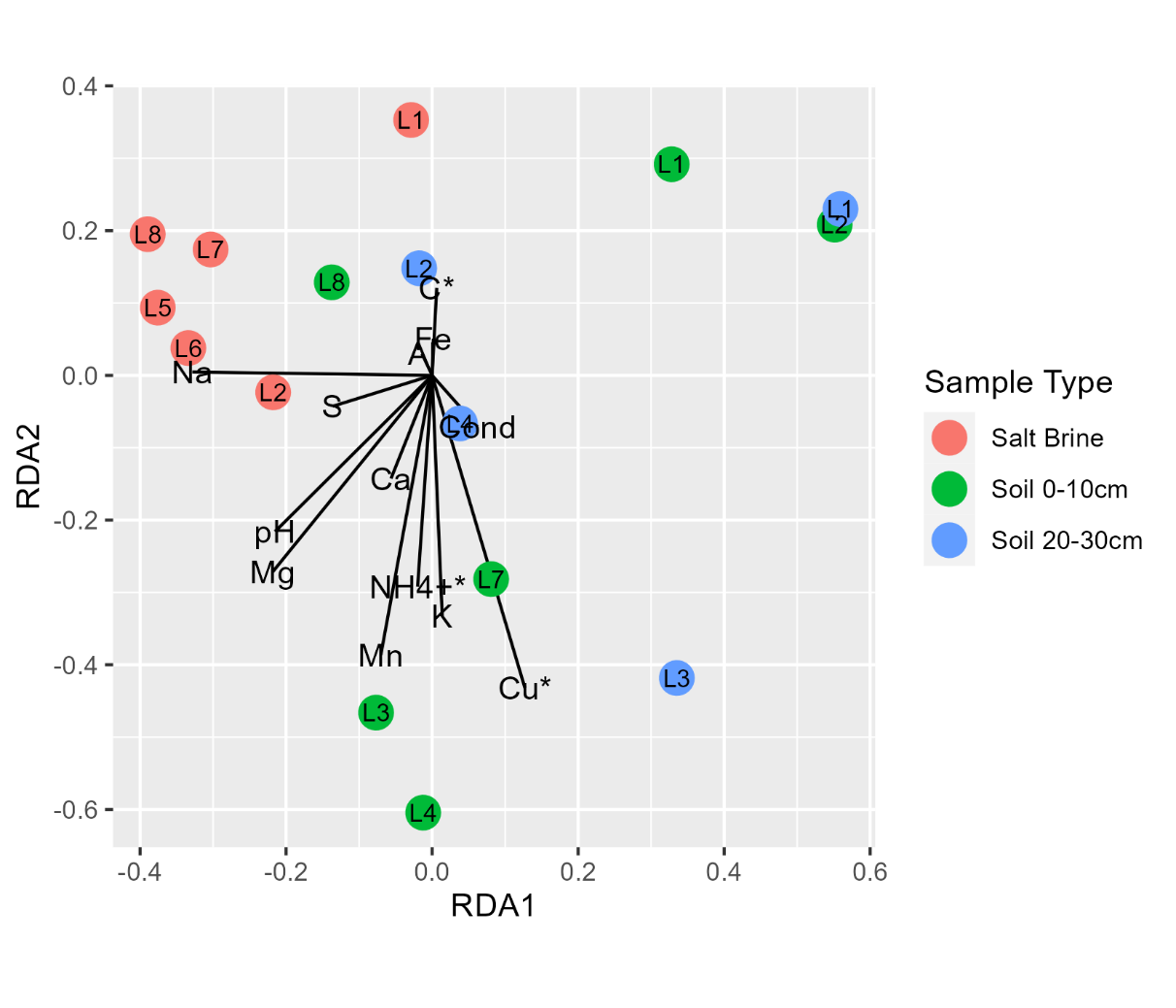


Figure 8: RDA analysis of the Fungal populations in soil and salt samples. Proportion explained, RDA1 15.5%, RDA2 11.3%. Adjusted R squared = 0.22. ANOVA testing of the model had a p value of 0.5. Environmental data that was significant in explaining variance by ANOVA is indicated by an asterisk.


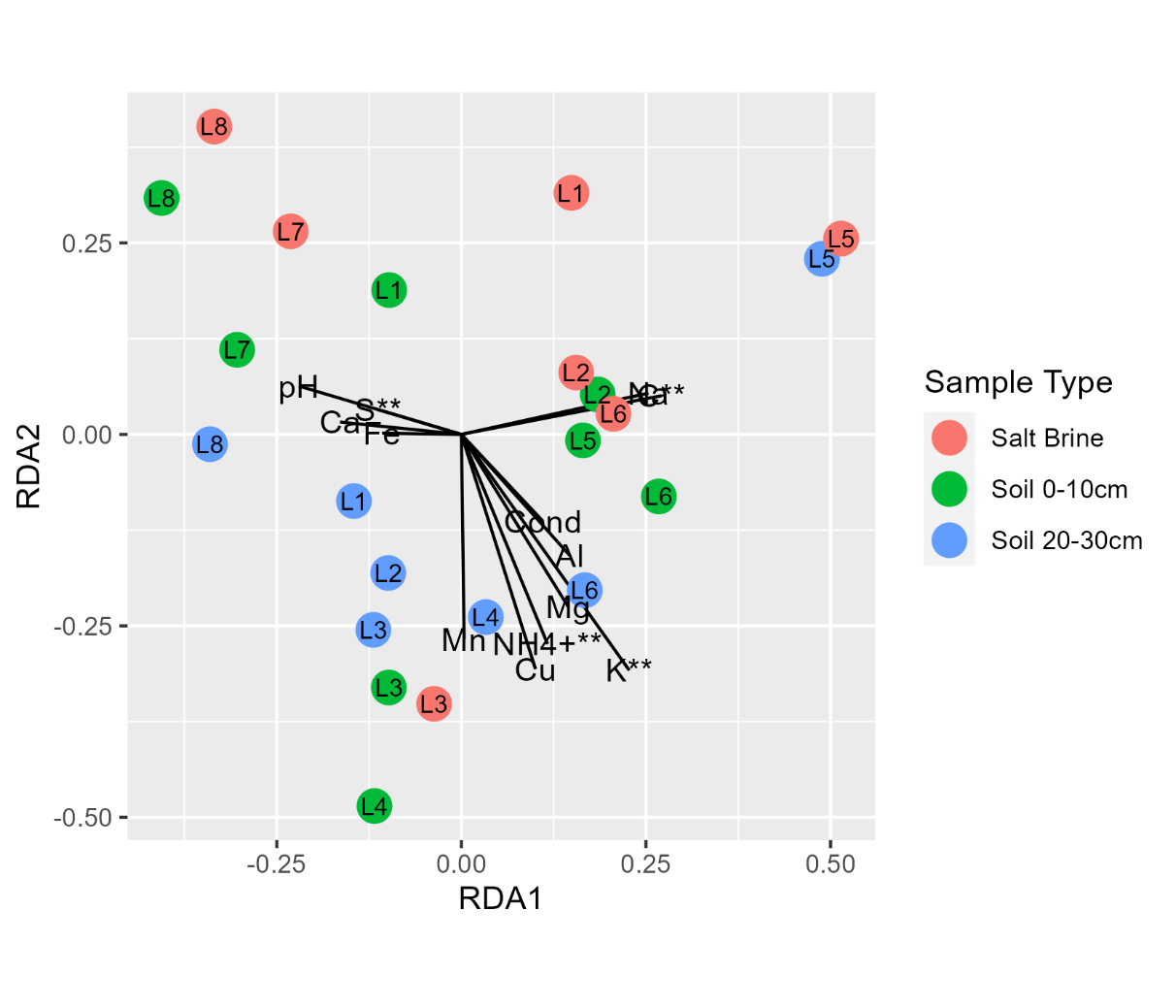


Figure 9: RDA analysis of the Eukarya populations in soil and salt samples. Proportion explained, RDA1 8.4%, RDA2 7.6%. Adjusted R squared = 0.08. ANOVA testing of the model had a p value of 0.009. Environmental data that was significant in explaining variance by ANOVA is indicated by an asterisk.
